# Smoc1 and Smoc2 regulate bone formation as downstream molecules of Runx2

**DOI:** 10.1038/s42003-021-02717-7

**Published:** 2021-10-19

**Authors:** Yoshifumi Takahata, Hiromasa Hagino, Ayaka Kimura, Mitsuki Urushizaki, Sachi Kobayashi, Kanta Wakamori, Chika Fujiwara, Eriko Nakamura, Kayon Yu, Hiroshi Kiyonari, Kana Bando, Tomohiko Murakami, Toshihisa Komori, Kenji Hata, Riko Nishimura

**Affiliations:** 1 Department of Molecular and Cellular Biochemistry, Osaka University Graduate School of Dentistry, Osaka, Japan; 2 Laboratory for Animal Resources and Genetic Engineering, RIKEN Center for Biosystems Dynamics Research, Hyogo, Japan; 3 Basic and Translational Research Center for Hard Tissue Disease, Nagasaki University Graduate School of Biomedical Sciences, Nagasaki, Japan

**Keywords:** Bone development, Bone

## Abstract

Runx2 is an essential transcription factor for bone formation. Although osteocalcin, osteopontin, and bone sialoprotein are well-known Runx2-regulated bone-specific genes, the skeletal phenotypes of knockout (KO) mice for these genes are marginal compared with those of Runx2 KO mice. These inconsistencies suggest that unknown Runx2-regulated genes play important roles in bone formation. To address this, we attempted to identify the Runx2 targets by performing RNA-sequencing and found *Smoc1* and *Smoc2* upregulation by Runx2. Smoc1 or Smoc2 knockdown inhibited osteoblastogenesis. *Smoc1* KO mice displayed no fibula formation, while *Smoc2* KO mice had mild craniofacial phenotypes. Surprisingly, *Smoc1* and *Smoc2* double KO (DKO) mice manifested no skull, shortened tibiae, and no fibulae. Endochondral bone formation was also impaired at the late stage in the DKO mice. Collectively, these results suggest that Smoc1 and Smoc2 function as novel targets for Runx2, and play important roles in intramembranous and endochondral bone formation.

## Abstract (web summary)

Takahata et al. investigate the functional role of SMOC1/2 proteins in skeletal development. They reveal a genetic pathway that includes Bmp2 and Runx2 inducing expression of the paralogous Smoc genes, which may offer novel and effective therapeutic strategies associated with various bone and cartilage diseases.

## Introduction

In vertebrates, two different types of bone-formation processes, intramembranous ossification and endochondral ossification, are known to occur during embryonic and postnatal skeletogenesis. In intramembranous ossification, mesenchymal stem cells directly differentiate into osteoblasts that subsequently form bone tissues^1,2^. For endochondral ossification, chondrocytes differentiate from mesenchymal stem cells to form cartilage tissues that are eventually replaced by bone tissues containing osteoblasts and osteoclasts^3,4^.

Bone morphogenetic protein (Bmp) family members are powerful cytokines that exhibit bone and cartilage formation activities by inducing osteoblast and chondrocyte differentiation. Among the Bmp family members, Bmp2 regulates the expressions and functions of runt-related transcription factor 2 (Runx2), Sp7 transcription factor 7 (Osterix), and Sex determining region Y-box 9 (Sox9), as critical transcription factors for bone and cartilage development^5–7^.

In particular, Runx2, a member of the Runt family of transcription factors, plays an indispensable role in bone formation and osteoblastogenesis. *Runx2*-deficient mice manifest no bone formation^8^. Mutations in the *RUNX2* gene cause cleidocranial dysplasia, characterized by impaired bone formation in the calvaria and clavicles^9^. Runx2 was also identified as a transcription factor that binds to the osteoblast-specific element 2 present in the osteocalcin (Ocn) gene promoter^10^. In addition, Runx2 was sufficient to promote mesenchymal cell differentiation into osteoblasts^11^. During osteoblast differentiation, Runx2 specifically regulated the expressions of osteoblast-specific and osteogenic genes, including Ocn, osteopontin, and bone sialoprotein^12–14^. However, the skeletal phenotypes in knockout (KO) mice for these genes are very marginal or absent compared with those in *Runx2* KO mice^14–18^. Therefore, it is predicted that currently unknown Runx2-target molecules play critical roles in bone formation.

In this study, we aimed to identify critical downstream molecules of Runx2 and investigate their functional roles in skeletal development. RNA-sequence and reverse transcription-quantitative polymerase chain reaction (RT-qPCR) analyses indicated that *Smoc1* and *Smoc2* were induced by both, Runx2 and Bmp2. Dominant-negative (DN)-Runx2 suppressed *Smoc1* and *Smoc2* expressions and their expressions were reduced in Runx2 KO mice. Smoc1 and Smoc2 knockdown inhibited mouse osteoblast differentiation and mineralization. Moreover, *Smoc1* and *Smoc2* double-knockout (DKO) mice manifested no skull formation, dwarfism, and shortened long bones. Histological analyses revealed impaired endochondral ossification at the late stage when Runx2 is required. Therefore, our results demonstrated that Smoc1 and Smoc2 function as transcriptional target modules and play important roles in bone formation.

## Results

### *Smoc1 and Smoc2* regulation by Runx2 and Bmp2

**Fig. 1:**
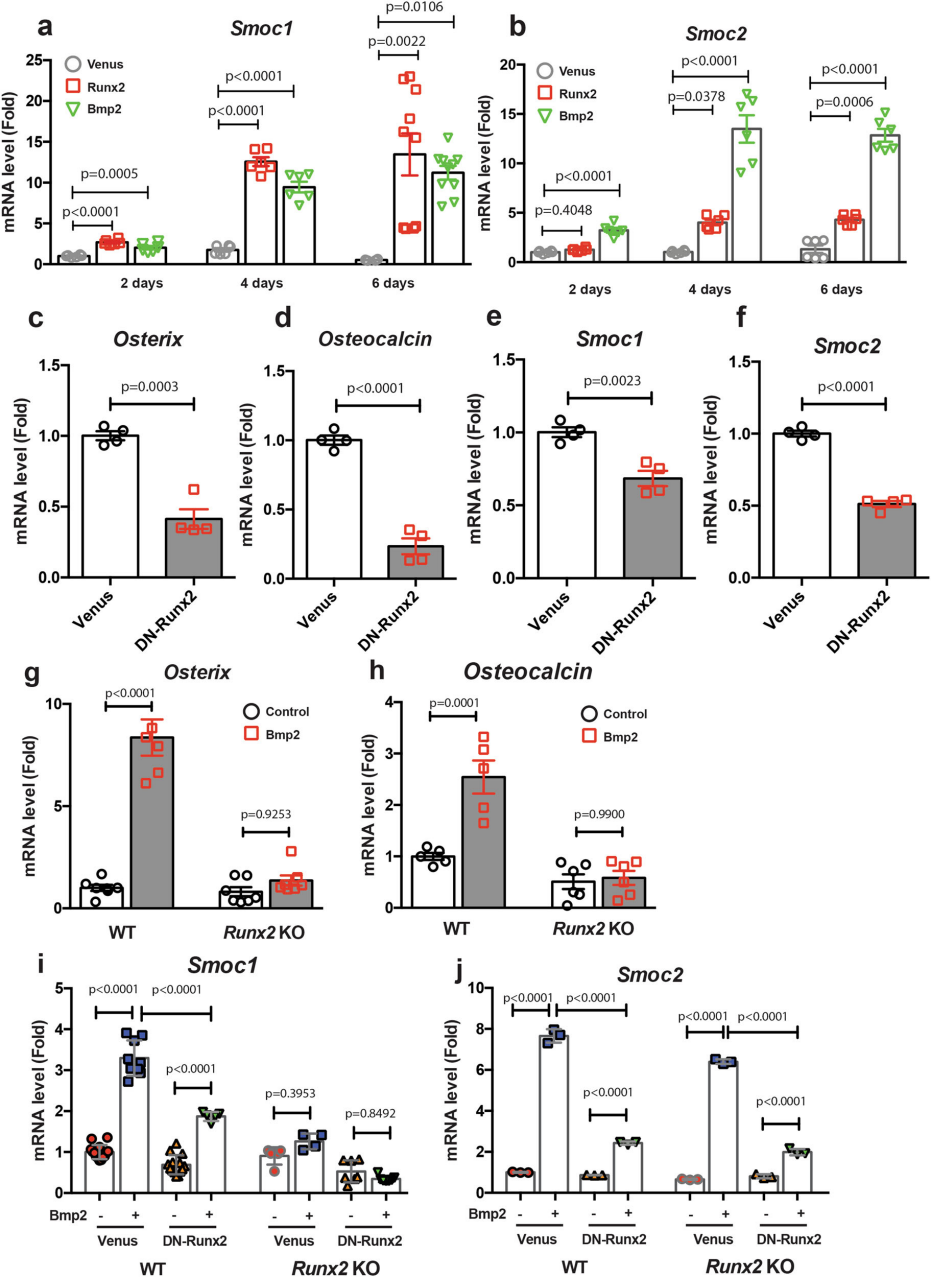
Regulation of *Smoc1 and Smoc2* expressions by Runx2 and Bmp2. **a**, **b** Primary limb bud cells were infected with Venus (control), Runx2, or Bmp2 adenoviruses. At 2, 4, or 6 days after infection, total RNA was extracted from the cells and *Smoc1* (**a**) and *Smoc2* (**b**) mRNA expressions were determined by RT-qPCR (*n* = 6–10, biologically independent samples). **c**–**f** Primary osteoblasts isolated from mouse calvariae were infected with Venus or DN-Runx2 adenoviruses. At 3 days after infection, total RNA was extracted and *Osterix* (**c**), *Osteocalcin* (**d**), *Smoc1* (**e**), and *Smoc2* (**f**) mRNA expressions were determined by RT-qPCR (*n* = 4, biologically independent samples). **g**, **h** Limb bud cells isolated from WT or *Runx2* KO littermate mice were incubated with or without Bmp2 (500 ng/mL) for 4 days. The total RNA was extracted from the cells and *Osterix* (**g**) and *Osteocalcin* (**h**) mRNA expressions were determined by RT-qPCR (*n* = 5–8, biologically independent samples). **i**, **j** Limb bud cells isolated from WT or *Runx2* KO littermate mice were infected with Venus or DN-Runx2 adenovirus, followed by incubation with or without Bmp2 (500 ng/mL). After 4 days of incubation, *Smoc1* (**i**), and *Smoc2* (**j**) mRNA expressions were determined by RT-qPCR (Smoc1: *n* = 14, Smoc2: *n* = 3, biologically independent samples). Values are the mean ± SE.

To identify important molecules involved in osteoblastogenesis and bone formation, we performed RNA-sequence analyses using limb bud cells infected with control or Runx2 adenoviruses, or Bmp2 adenovirus, which regulates Runx2 function and expression^19^ and induces bone formation. Searching for genes that displayed a log2 fold-change value of >1 expression level in cells infected with Runx2 or Bmp2, we determined the upregulation of 653 genes by Bmp2 and 497 genes by Runx2 (Supplementary Fig. 1 and Supplementary Data 2 and 3). From the 180 common genes that were upregulated by both, Bmp2 and Runx2 (Supplementary Fig. 1d), we focused on the matrix proteins Smoc1 and Smoc2, which contain an extracellular calcium-binding domain^20,21^, and are members of the secreted acidic cysteine-rich glycoprotein (SPARC)-related family genes. We confirmed the induction of *Smoc1* and *Smoc2* expressions in limb bud cells by both, Runx2 and Bmp2 by RT-qPCR analyses (Figs. 1a, b). To understand the importance of Runx2 for *Smoc1* and *Smoc2* expressions, we determined the effects of DN-Runx2 on their expressions in primary osteoblasts isolated from mouse calvariae. As expected, DN-Runx2 overexpression markedly inhibited *Osterix* and *Osteocalcin* expressions, both of which are well-known Runx2 transcriptional targets^19,22^ in primary osteoblasts (Fig. 1c, d). *Smoc1* and *Smoc2* expressions were significantly inhibited by DN-Runx2 overexpression (Fig. 1e, f). To further assess the role of Runx2 in *Smoc1* and *Smoc2* regulation, we examined the effects of Bmp2 on limb bud cells prepared from *Runx2*-deficient and wild-type (WT) littermate mice. Sustained exposure of limb bud cells to Bmp2 induced *Runx2* expression (Supplementary Fig. 2), and Bmp2 induced *Osterix* and *osteocalcin* expressions in WT limb bud cells, but not in *Runx2* KO limb bud cells (Fig. 1g, h). DN-Runx2 treatment suppressed Bmp2-dependent *Smoc1* and *Smoc2* induction in WT limb bud cells (Fig. 1i). In addition, Bmp2-dependent *Smoc1* induction was absent in *Runx2* KO limb bud cells (Fig. 1i). By contrast, Bmp2 still induced *Smoc2* expression in *Runx2* KO limb bud cells. (Fig. 1j) This result suggests that a Runx2-independent Bmp2 signal is also involved in *Smoc2* upregulation. Indeed, Bmp2 and Runx2 synergistically induced *Smoc1* and *Smoc2* expressions as an early response to infection (Supplementary Fig. 3a, b). Furthermore, we confirmed Runx2 binding on the *Smoc1* and *Smoc2* genes promoters as determined by chromatin immunoprecipitation assays (Supplementary Fig. 4), referred to public ChIP-seq datasets^23^. These results suggest that Smoc1 and Smoc2 are downstream molecules of Runx2 and Bmp2 signaling.

### Smoc1 and Smoc2 expressions in skeletal tissues

**Fig. 2:**
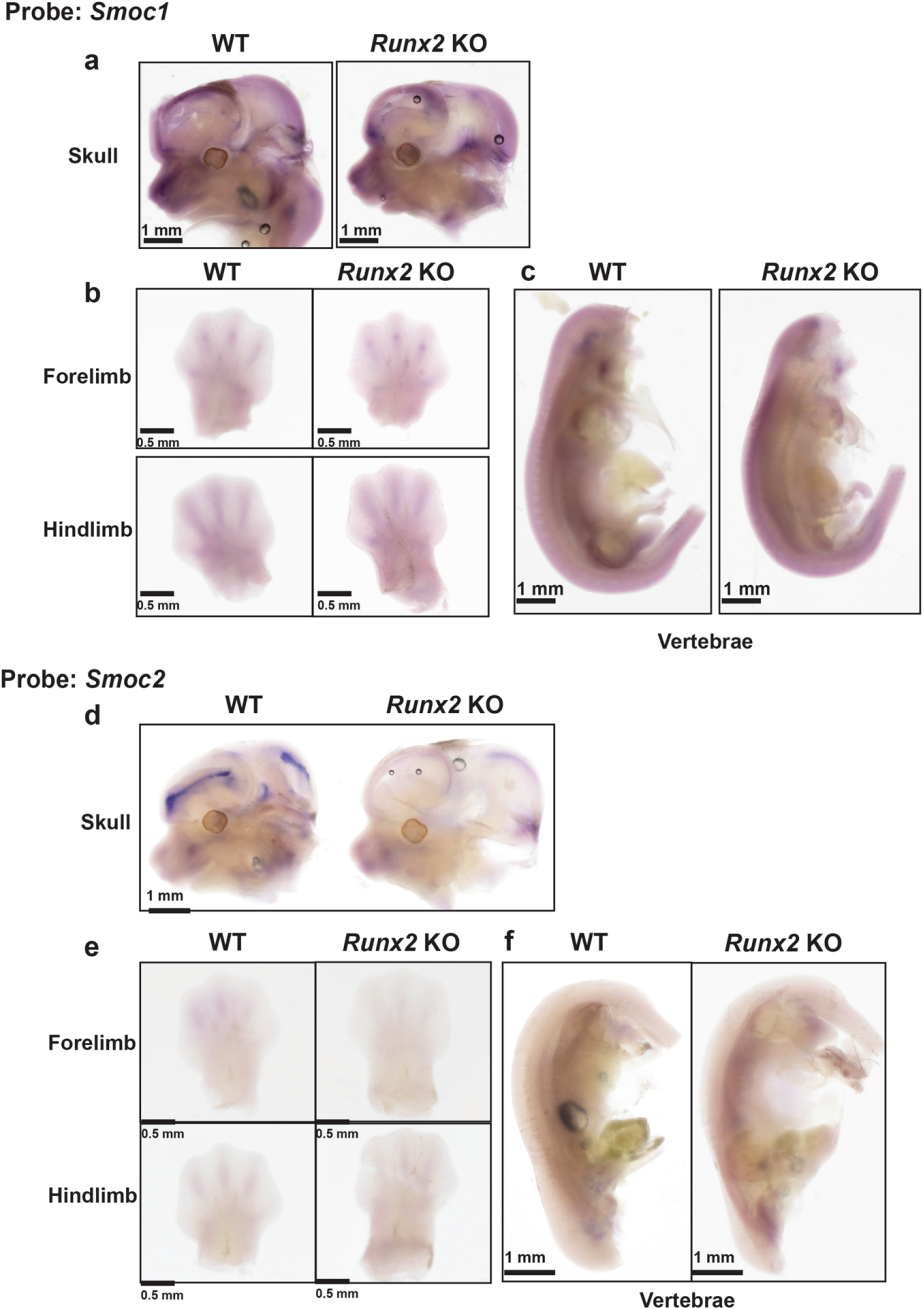
Expression of *Smoc1* and *Smoc2* in skeletal tissues. Whole-mount in situ hybridization analyses of (**a**) skull, (**b**) forelimb and hindlimb, and (**c**) vertebrae in *Runx2* KO and WT littermate mice at E12.5 for *Smoc1*. Whole-mount in situ hybridization analyses of (**d**) skull, (**e**) forelimb and hindlimb, and (**f**) vertebrae in *Runx2* KO or WT littermate mice at E12.5 for *Smoc2* (*n* = 4, biologically independent animals).

To clarify whether *Smoc1* and *Smoc2* expressions are regulated by Runx2 in vivo, we examined their expressions in *Runx2* KO and WT littermate mice at the E12.5 stage as determined by whole-mount in situ hybridization. The analyses revealed that *Smoc1* was strongly expressed in the skull (Fig. 2a), forelimbs, hindlimbs (Fig. 2b), and vertebrae (Fig. 2c). The *Smoc1* signals in the skull and vertebrae of *Runx2* KO mice were moderately less than those of WT mice (Fig. 2a, c). Strong *Smoc2* expression was observed in the skull of WT mice (Fig. 2d), whereas only very weak signals were detected in other tissues (Fig. 2e, f). Notably, a drastic decrease of *Smoc2* expression in the skull of *Runx2* KO mice was seen compared with that in the skull of WT mice (Fig. 2d). In addition, the localization of *Runx2* expression was similar to *Smoc2* expression in the skull (Supplementary Fig. 5a, b). These results suggest that Smoc1 and Smoc2 are downstream molecules of Runx2 in vivo.

### Roles of Smoc1 and Smoc2 in osteoblastogenesis

**Fig. 3:**
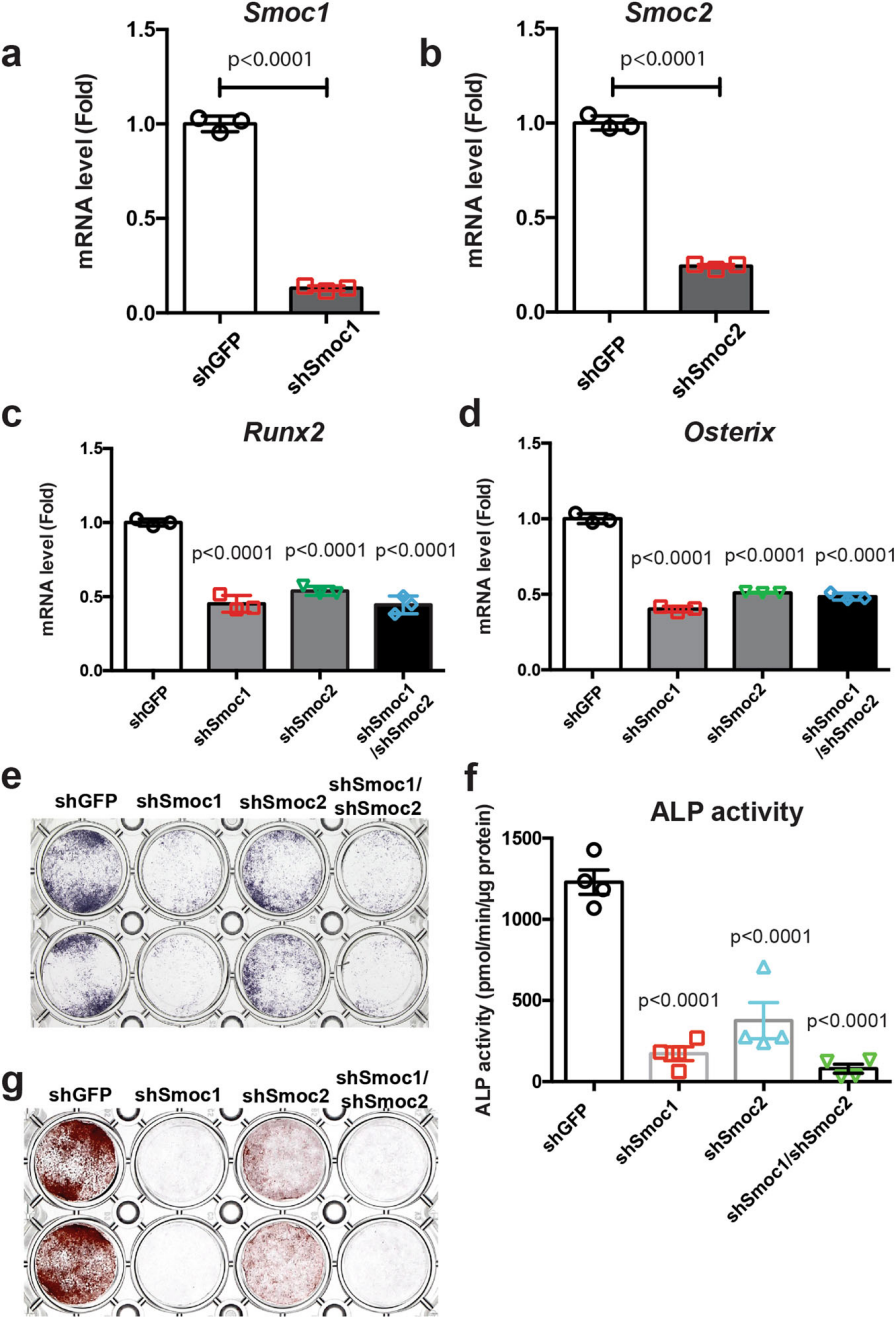
Roles of Smoc1 and Smoc2 in osteoblast differentiation. **a**–**d** Primary osteoblasts isolated from mouse calvariae were infected with shGFP, shSmoc1, shSmoc2, or both shSmoc1 and shSmoc2 retroviruses. At 72 h after infection, the culture medium was replaced with a new medium and the cells were infected with Bmp2 adenovirus for 6 h, followed by incubation in a differentiation medium containing 50 μg/mL ascorbic acid and 5 mM sodium β-glycerophosphate for 5 days. Total RNA was extracted from the cells and expressions of *Smoc1* (**a**), *Smoc2* (**b**), *Runx2* (**c**), and *Osterix* (**d**) mRNAs were determined by RT-qPCR. Values are the mean ± SE (*n* = 3, biologically independent samples). **e**–**g** Primary osteoblasts isolated from mouse calvariae were infected with shGFP, shSmoc1, shSmoc2, or both shSmoc1 and shSmoc2 retroviruses. The cells were infected with Bmp2 adenovirus and incubated in the presence of 50 μg/mL ascorbic acid and 5 mM sodium β-glycerophosphate. After incubation for 3 or 5 days, the cells were evaluated by alkaline phosphatase staining (*n* = 3, independent experiments) (**e**), alkaline phosphatase activity (*n* = 4, biologically independent samples) (**f**), and alizarin red staining (**g**) (*n* = 3, independent experiments).

To examine the functional roles of Smoc1 and Smoc2 in osteoblast differentiation, we performed knockdown experiments by employing short hairpin RNA (shRNA) approaches in primary osteoblasts isolated from mice calvariae. shRNA retrovirus infection against *Smoc1* (shSmoc1) and *Smoc2* (shSmoc2) efficiently inhibited *Smoc1* and *Smoc2* expressions in osteoblasts, respectively (Fig. 3a, b). *Runx2* and *Osterix* expressions were significantly suppressed in osteoblasts by shSmoc1 and/or shSmoc2 retrovirus infection (Fig. 3c, d). Furthermore, shSmoc1 and/or shSmoc2 retrovirus infection inhibited osteoblast alkaline phosphatase activity (Fig. 3e, f) and mineralization (Fig. 3g). These data suggest that both, Smoc1 and Smoc2, play a role in osteoblast differentiation and maturation.

### Roles of Smoc1 and Smoc2 in bone and cartilage development

**Fig. 4:**
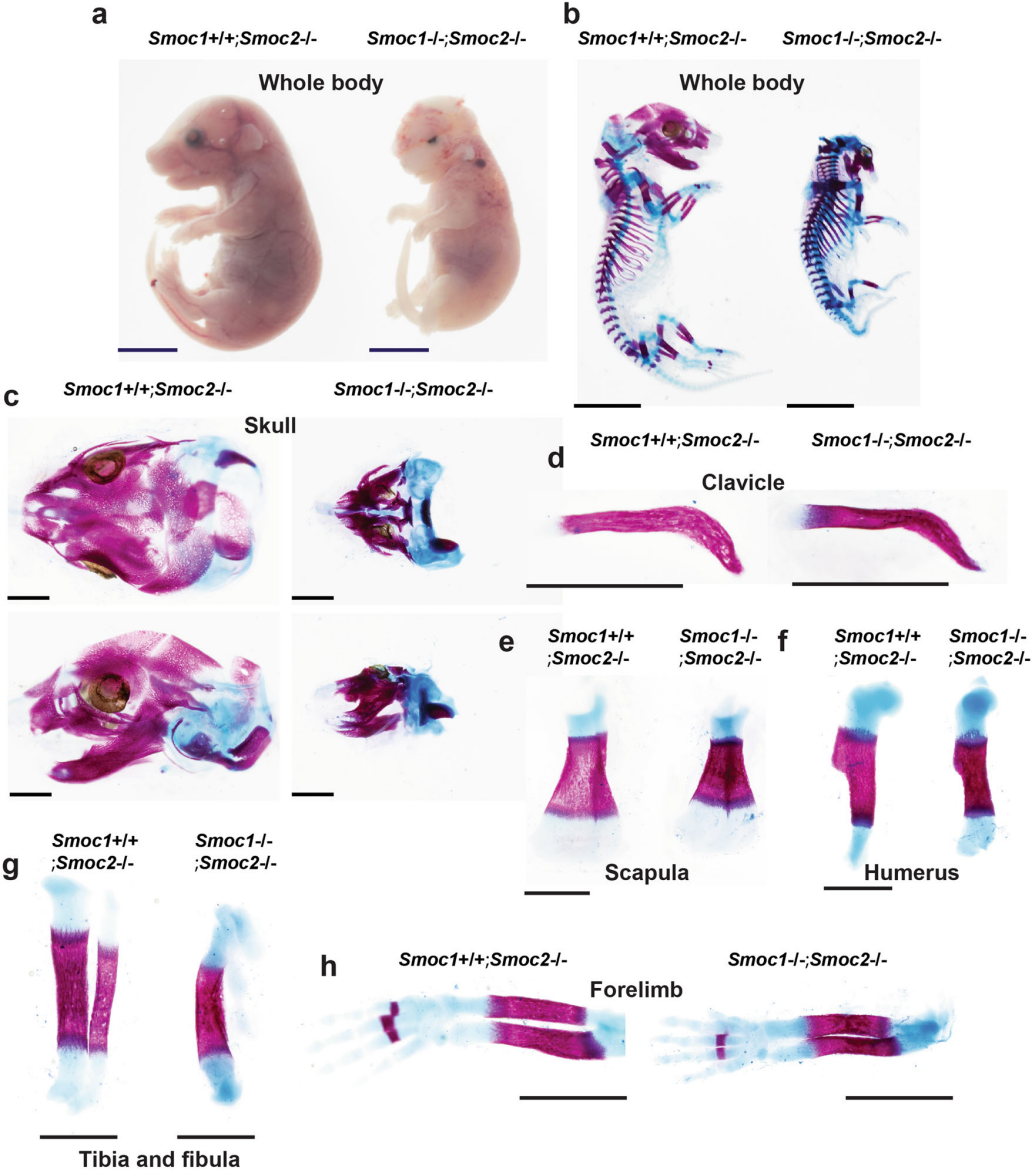
Skeletal abnormalities in *Smoc1/2* DKO mice. *Smoc1*^+/−^;*Smoc2*^−/−^ mice were mated, and *Smoc1*^+/+^;*Smoc2*^−/−^ and *Smoc1*^−/−^;*Smoc2*^−/−^ littermate mice were analyzed at E17.5 macroscopically under a stereoscopic microscope (**a**) and by skeletal preparations stained with alizarin red and alcian blue (**b**–**h**). **a** Growth retardation in *Smoc1*^−/−^;*Smoc2*^−/−^ mice. Staining in the whole body (**b**), skull (**c**), clavicle (**d**), scapula (**e**), humerus (**f**), tibia and fibula (**g**), and forelimb (**h**) of *Smoc1*^+/+^;*Smoc2*^−/−^ and *Smoc1*^−/−^;*Smoc2*^−/−^ littermate mice. **a**, **b** Scale bars: 5 mm. **c**–**h** Scale bars: 1 mm.

To evaluate the functional roles of Smoc1 and Smoc2 in bone formation in vivo, we generated *Smoc1* and *Smoc2* KO mice (Supplementary Figs. 6 and 7). To generate *Smoc1* KO mice, we first generated *Smoc1* flox mice and mated these with CAG-Cre transgenic mice. Following the generation of *Smoc1* heterozygous deficient mice, we established *Smoc1* KO mice by mating the heterozygous mice (Supplementary Fig. 6). Okada et al. previously reported that *Smoc1* homozygous mutant mice displayed a phenotype of ocular dysplasia, limb malformation, and fibula loss^24^. These *Smoc1* KO mice were viable at postnatal day 0 (P0) but died within 3 weeks. Rainger et al. also showed that *Smoc1* mutant mice have defects of eye development, mispositioned femur and fibula, and hindlimb malformation^25^. Our *Smoc1* KO mice had a similar skeletal phenotype to these, however, they died shortly after birth, not surviving for more than a day (Supplementary Fig. 8); although we do not know the reason why our *Smoc1* KO mice are lethal. A recent study by Marchant et al. reported that canine brachycephaly is associated with the *SMOC2* gene function and that the dogs in which the *SMOC2* gene was mutated displayed dysmorphic skulls^26^. Our *Smoc2* KO mice showed almost normal growth and fertility (Supplementary Fig. 9a–f). However, a moderate craniofacial phenotype, with shorter nasal to the eye and nasal to parietal bone distances, was observed in *Smoc2* KO mice (Supplementary Fig. 9g–i). Because Smoc1 and Smoc2 have been proposed to functionally compensate for one another based on their highly conserved homology^20^, we generated DKO mice deficient in both *Smoc1* and *Smoc2* and assessed their phenotypes. Based on our observation that *Smoc2*^−/−^ mice had only marginal phenotypes in skeletal tissues compared with their WT littermates (Supplementary Fig. 9), we first mated *Smoc1*^+/−^;*Smoc2*^−/−^ mice with each other and used the *Smoc2*^−/−^ littermate mice as control mice for the *Smoc1* and *Smoc2* DKO mice. Deletion of the *Smoc1* or *Smoc2* gene did not affect the expression level of the other gene in vivo (Supplementary Fig. 5b, c). A severe craniofacial phenotype, which resulted in neonatal lethality of the mice, and moderate dwarfism were observed in *Smoc1*^−/−^;*Smoc2*^−/−^ mice (Fig. 4a–c), whereas clavicle formation appeared normal in Smoc1^−/−^;*Smoc2*^−/−^ mice and *Smoc2*^−/−^ mice at E17.5 (Fig. 4d). Shortened scapula, humerus, tibia, and forelimb were observed in *Smoc1*^−/−^;*Smoc2*^−/−^ mice compared with *Smoc2*^−/−^ mice (Fig. 4e–h). The tibia in *Smoc1*^−/−^;*Smoc2*^−/−^ mice was also bent (Fig. 4g). Cranial hypoplasia was also confirmed in the earlier developmental stages of E12.5 and E13.5 (Supplementary Fig. 10). To further analyze the skeletal phenotype of *Smoc1* and *Smoc2* DKO mice, we next mated *Smoc1*^+/−^;*Smoc2*^+/−^ mice. As a result of counting all the genotyping patterns of the E18.5 offspring, the number of individuals obtained was approximately according to Mendel’s laws (Supplementary Data 4). Consistent with the results shown in Fig. 4, *Smoc1* and *Smoc2* DKO mice displayed dwarfism, complete loss of calvariae, skull hypoplasia, and shortened limbs (Supplementary Fig. 11). Interestingly, *Smoc1*^−/−^;*Smoc2*^+/−^ mice also showed severely deficient calvariae morphogenesis and exhibited craniofacial anomalies (Supplementary Fig. 11 and Supplementary Data 4). These results indicate that Smoc1 and Smoc2 play important roles in bone formation and craniofacial development.

Our *Smoc1* KO mice displayed loss of the fibulae and consistent phenotypes with *Smoc1*^tm1a/tm1a^ mutant mice (Supplementary Fig. 8). Because it has been reported that fibula loss is caused by *Homeobox C11* (*Hoxc11*) gene overexpression^27^, we examined the involvement of Hoxc11 in the phenotype observed in *Smoc1* KO mice. Interestingly, *Hoxc11* expression was significantly upregulated by Bmp2 (Supplementary Fig. 12a). We also found an association of Hoxc11 with Runx2 by performing co-immunoprecipitation experiments (Supplementary Fig. 12b). However, Hoxc11 overexpression did not affect *Smoc1* or *Smoc2* expressions (Supplementary Fig. 12c, d). *Runx2*, *Osterix*, and *osteocalcin* expressions were unaltered by Hoxc11 overexpression (Supplementary Fig. 12e–g). Taken together, it is unlikely that Hoxc11 plays a role in fibula formation through Smoc1 or Smoc2, and other molecules would be involved in Bmp2-induced *Smoc1* and *Smoc2* expressions.

**Fig. 5:**
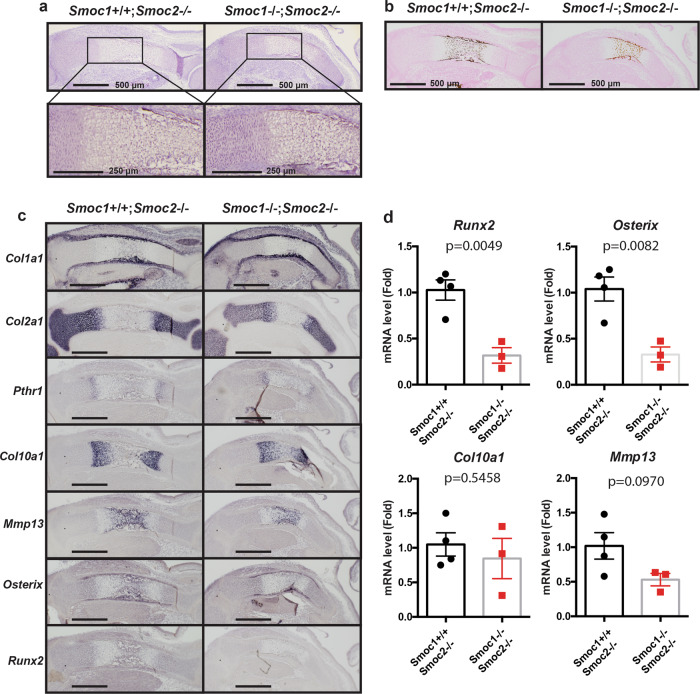
Delayed endochondral bone formation in *Smoc1/2* DKO mice. *Smoc1*^+/−^;*Smoc2*^−/−^ mice were mated, and *Smoc1*^+/+^;*Smoc2*^−/−^ and *Smoc1*^−/−^;*Smoc2*^−/−^ littermate mice at E15.5 were examined by hematoxylin-eosin (**a**) and von Kossa (**b**) staining (*n* = 4). **c** In situ hybridization analyses using antisense probes against *Col1a1*, *Col2a1*, *Pthr1*, *Col10a1*, *Mmp13*, *Osterix*, and *Runx2*. Scale bars: 500 μm (*n* = 4, biologically independent animals). **d** The total RNA was extracted from the tibia of *Smoc1*^+/+^;*Smoc2*^−/−^ or *Smoc1*^−/−^;*Smoc2*^−/−^ littermate mice at E15.5. The *Runx2*, *Osterix*, *Col10a1*, and *Mmp13* expression levels were determined by RT-qPCR (*Smoc1*^+/+^;*Smoc2*^−/−^: *n* = 4, *Smoc1*^−/−^;*Smoc2*^−/−^: *n* = 3, biologically independent animals). Values are the mean ± SE.

**Fig. 6:**
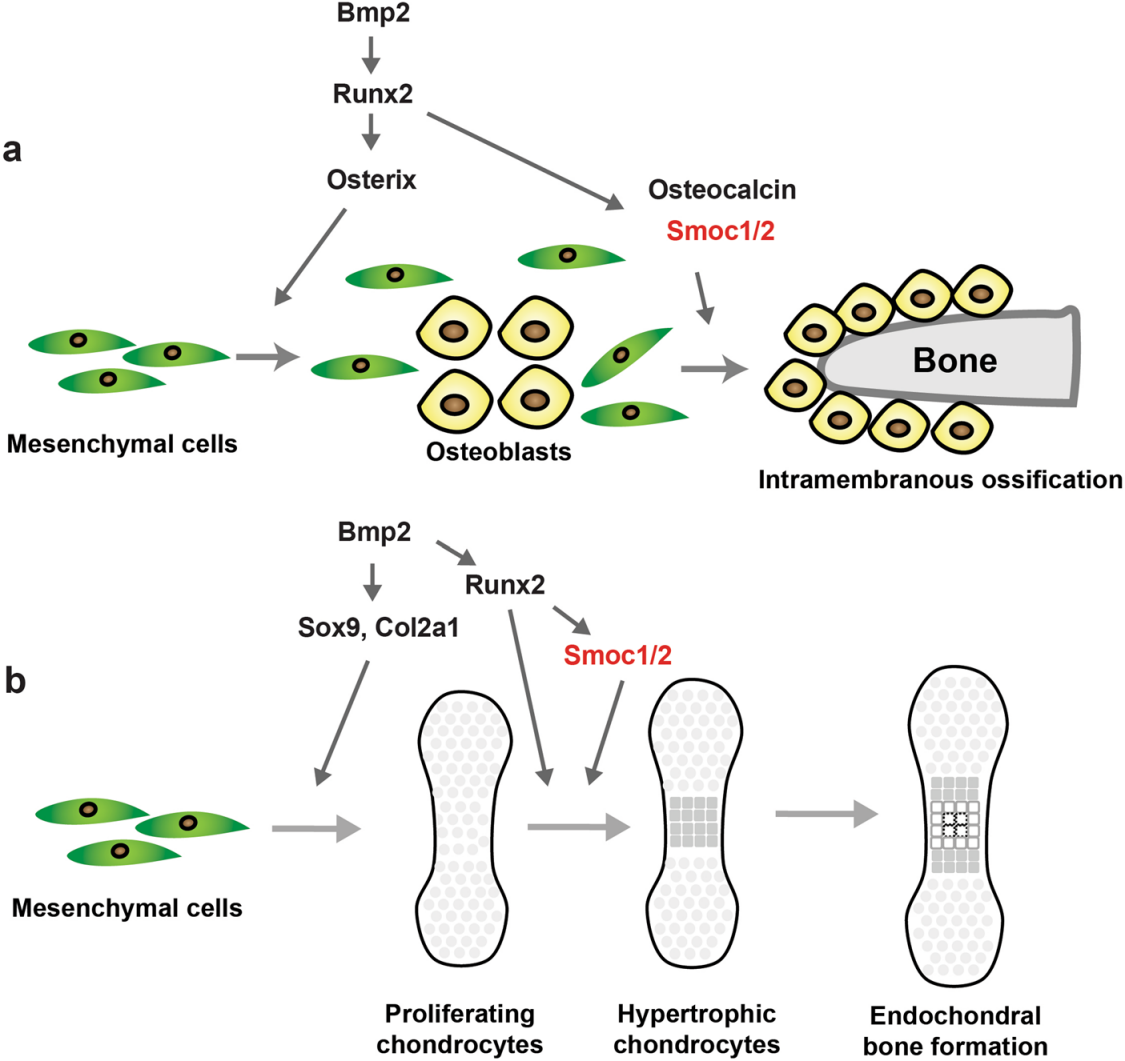
Schematic model of the Bmp2–Runx2–Smoc1/2 axis in bone formation. **a** For intramembranous ossification, the Bmp2–Runx2 axis directly induces Smoc1 and Smoc2; Smoc1 and Smoc2 play pivotal roles in bone formation. **b** The Bmp2–Sox9 axis regulates Col2a1 expression and the early stage of endochondral ossification. The Bmp2–Runx2–Smoc1/2 axis regulates the hypertrophic stage of endochondral ossification.

Because we observed dwarfism and shortened long bones in *Smoc1* and *Smoc2* DKO mice (Fig. 4 and Supplementary Figs. 10 and 11), we investigated the roles of Smoc1 and Smoc2 in endochondral bone formation. To achieve this, we histologically examined the tibia in *Smoc1* and *Smoc2* DKO mice at E15.5. Endochondral ossification appeared delayed in *Smoc1* and *Smoc2* DKO mice compared with *Smoc2* KO mice (Fig. 5a, b). *Collagen, type I, alpha 1 (Col1a1)*, *Collagen, type II, alpha 1* (*Col2a1)*, and *Parathyroid hormone 1 receptor* expressions appeared normal in *Smoc1* and *Smoc2* DKO mice (Fig. 5c). *Col10a1* expression was clearly separated from diaphysis in *Smoc2* KO mice, however, it was not still separated in the diaphysis of *Smoc1* and *Smoc2* DKO mice, supporting the notion that the late stage of endochondral bone formation was delayed in the DKO mice (Fig. 5c, d). Similarly, *Matrix metallopeptidase 13* (*Mmp13)*, *Osterix*, and *Runx2* expressions were reduced in *Smoc1* and *Smoc2* DKO mice (Fig. 5c, d). These data demonstrate that Smoc1 and Smoc2 are required for the late stage of endochondral bone formation, during which step Runx2 plays an important role (Fig. 6).

We subsequently attempted to examine whether Smoc1 and Smoc2 play a role in the differentiation of mesenchymal cells to osteoblasts and/or chondrocytes. To address this, we examined the effect of Smoc1 and Smoc2 on the expressions of osteoblast and chondrocyte marker genes in Bmp2-stimulated limb bud cells isolated from *Smoc1* or *Smoc2* KO and WT littermate mice. The early chondrocyte markers, *Col2a1* and *Sox9*, were decreased in the *Smoc1*-deficient limb bud cells (Supplementary Fig. 13a), but not in the *Smoc2*-deficient limb bud cells (Supplementary Fig. 14a, b). Similar expression patterns were observed for *Mmp13* (Supplementary Figs. 13a and 14f). In addition, *Smoc1* deficiency decreased *Osterix* and *Runx2* expressions (Supplementary Fig. 13a), both of which are essential transcription factors for mesenchymal cell differentiation to osteoblasts, whereas *Smoc2* deficiency did not affect *Runx2* or *Osterix* expressions (Supplementary Fig. 14d, e). These results suggest that Smoc1 might be involved in mesenchymal cell differentiation to osteoblasts and chondrocytes, but not Smoc2. The findings were supported by the results that chondrocyte and osteoblast differentiation were suppressed in *Smoc1*-deficient cells as determined by alcian blue staining and alkaline phosphatase (ALP) activity assays, respectively (Supplementary Fig. 13b, c). These results suggest that Smoc1 and Smoc2 have different effects on osteoblast and chondrocyte differentiation from mesenchymal cells.

## Discussion

In this study, we attempted to identify Runx2-target molecules involved in bone formation and isolated Smoc1 and Smoc2 as such molecules. Smoc1 and Smoc2, are members of the SPARC/osteonectin family, are matricellular proteins associated with basement membranes, and are characterized by the presence of an extracellular domain and a follistatin-like domain^28^. Osteonectin is predominantly expressed in osteoblasts, and participates in the regulation of cell–matrix interactions, consequently influencing bone mineralization and angiogenesis^29–31^. However, *osteonectin* KO mice appeared normal and fertile until 6 months of age and had no obvious phenotypes in their skeletal tissues^32,33^. Although *osteonectin* was not increased by Bmp2 treatment, *Smoc1* and *Smoc2* were upregulated by both, Runx2 and Bmp2 (Fig. 1). Consistently, DN-Runx2 overexpression significantly inhibited Bmp2-induced *Smoc1* and *Smoc2* expressions. Moreover, Bmp2 failed to upregulate *Smoc1* expression in *Runx2*-deficient limb bud cells. Importantly, Bmp2-induced osteoblast differentiation of primary osteoblasts was clearly suppressed by Smoc1 or Smoc2 knockdown. In addition, *Smoc1* and *Smoc2* were highly expressed in mouse embryo calvariae and limbs (Fig. 2). Taken together, these results indicate that the SPARC family matrix proteins, *Smoc1* and *Smoc2*, are expressed in skeletal tissues, particularly the skull, function as important downstream molecules of Runx2, and play roles in osteoblastogenesis (Fig. 6).

Mutations in the *SMOC1* gene were shown to result in ophthalmo-acromelic syndrome (OAS), also known as Waardenburg anophthalmia syndrome, which manifests a distinctive pattern of distal limb anomalies^34,35^. Genetically constructed *Smoc1* mutant mice by gene-trapped technology, *Smoc1*^tm1a/tm1a^, with disruption of *Smoc1* gene expression to ~10% of the normal level, displayed similar malformation of the hindlimbs to that observed in the limbs of human OAS patients^25^. Consistently, our *Smoc1* KO mice showed similar skeletal phenotypes to *Smoc1*^tm1a/tm1a^ mice (Supplementary Fig. 8).

Because the homology between Smoc1 and Smoc2 is very high and their expressions coincide in skeletal tissues, we hypothesized that Smoc1 and Smoc2 functionally compensate one another during bone formation. Interestingly, *Smoc1* and *Smoc2* DKO mice displayed severe phenotypes in skeletogenesis, including the complete loss of calvariae, ocular dysplasia, and shortened limbs. In addition, *Smoc1*^+/−^;*Smoc2*^−/−^ mice showed no skull formation. Considering that *Smoc2*^−/−^ mice appeared normal skull formation, it would be possible that *Smoc2* deficiency might not be essential for skull formation. However, consistent with the previous literature (26), we observed mild skull dysplasia in *Smoc2* KO mice (Supplementary Fig. 9). Therefore, dosages and different tissue expressions of *Smoc1* and *Smoc2* are closely associated with the different bone phenotypes observed in the *Smoc1* and *Smoc2* mutant mice. In addition, histological analyses of *Smoc1* and *Smoc2* DKO mice revealed delayed endochondral ossification at the late stage, when Runx2 is functional. Taken together, these results indicate that Smoc1 and Smoc2 compensate each other and play important roles in both intramembranous and endochondral bone formation. By contrast, clavicle formation appeared normal in *Smoc1* and *Smoc2* DKO mice compared with control mice. We speculate that the normal clavicle formation in *Smoc1* and *Smoc2* DKO mice was due to low *Smoc1* and *Smoc2* expression levels in the clavicle and/or the involvement of other molecules.

There were differences in phenotypic severity, particularly in the extremity bones during endochondral bone formation. We observed that fibular hypoplasia and tibial bending were present in *Smoc1* and *Smoc2* DKO mice, whereas there was little morphological difference in the humerus, scapula, and forelimb between *Smoc1* and *Smoc2* DKO mice and their control littermate mice (Fig. 4). Furthermore, toe fusion was observed in the hindlimb, but not in the forelimb. It is speculated that Smoc1 and Smoc2 may have unknown functions such as interactions with molecules involved in patterning and development associated with the determination of the forelimbs and hindlimbs. Similarly, we observed different expression patterns between Smoc1 and Smoc2 in the developmental stage (Fig. 2 and Supplementary Fig. 5). These findings suggested that different Bmp ligands temporally and spatially regulate Smoc1 and Smoc2 in several tissues, including the eye (25).

Functional studies needed to understand the SMOC family still appear complex. Restricted distribution of Bmp signaling is regulated by a number of mechanisms, as shown in previous reports; ligand binding by extracellular Bmp antagonists^36^; interactions with extracellular matrix proteins, including heparan sulfate proteoglycans^37^. Smoc protein has also been shown to bind to heparan sulfate proteoglycans^38^. Smoc1 has been shown to inhibit Bmp signaling and to be essential for postgastrulation development in *Xenopus*^39^. Application of Bmp2 to NIH3T3 fibroblasts transfected with SMOC was shown to inhibit Smad1/5/8 phosphorylation^39^. *Pent*, the orthologue pentagon of *Smoc* in *Drosophila*, was found to be expressed in developing wing imaginal discs and to inhibit BMP signaling^40^. Meanwhile, a recent study showed that SMOC proteins have dual functions as BMP inhibitors and expanders of BMP signaling^41^. Therefore, Smoc functions may possibly play a role in regulating Bmp2 signaling, however, to the best of our knowledge, there have been no reports of Smoc functions in mammals. Because the precise molecular mechanisms by which Smoc1 and Smoc2 regulate bone and cartilage formation remain unclear in the present study, further detailed analyses are required in future studies. We believe that a better understanding of the molecular mechanisms could be achieved through the identification of the cell-surface receptors for Smoc1 and Smoc2. Thus, our findings reveal that the Bmp2–Runx2–Smoc1/Smoc2 axis plays an important role in bone formation; it may offer novel and effective therapeutic strategies associated with various bone and cartilage diseases.

## Methods

### Cells and reagents

LentiX-293T cells were purchased from Takara (Shiga, Japan). Plat-E cells were a generous gift from Dr. Kitamura (The University of Tokyo, Tokyo, Japan). Osteoblasts and limb bud cells were cultured in alpha modification of Eagle’s minimum essential media (α-MEM; Thermo Fisher, Waltham, MA, USA) containing 10% fetal bovine serum (FBS) at 37 °C in a humidified 5% CO_2_ incubator. Plat-E cells and LentiX-293T cells were cultured in Dulbecco’s modified Eagle’s medium (DMEM; Thermo Fisher) containing 10% FBS. Recombinant Bmp2 was obtained from a conditioned medium of LentiX-293T cells transfected with a Bmp2 expression vector as described previously^42^. Bmp2 activity was determined by comparison with human recombinant Bmp2 (Peprotech, Rocky Hill, NJ, USA).

Osteoblasts were isolated from calvariae of 3–5-day-old neonatal mice by a sequential enzymatic digestion method as described previously^43^. Briefly, mouse calvariae were gently incubated with 5 mM ethylenediaminetetraacetic acid (EDTA) in phosphate-buffered saline (PBS) for 1 h at 37 °C, followed by three 20-min digestions with 0.25% collagenase in DMEM for 20 min at 37 °C. Cells obtained during the last two digestion processes were collected together in α-MEM containing 10% FBS. Throughout the subsequent experiments, α-MEM containing 10% FBS, 50 μg/mL ascorbic acid, and 5 mM sodium β-glycerophosphate were used to induce osteoblastic differentiation. Limb bud cells were isolated from mouse embryos at E12–E13 and digested with 0.05% trypsin/0.53 mM EDTA in PBS for 10 min at 37 °C. Cells obtained during the digestion were collected in α-MEM containing 10% FBS. For monolayer culture, limb bud cells were seeded at 1.6 × 10^5^ cells/cm^2^. All other chemicals used were of the highest purity commercially available.

### RNA-sequence and data analysis

Limb bud cells were infected with Venus, Runx2, or Bmp2 adenovirus. After 4 days of incubation, total RNA was extracted using a Nucleospin RNA Plus Kit (Takara). Sequencing was performed on an Illumina HiSeq 2500 platform (Illumina, San Diego, CA, USA) in a 75-base single-end mode. Sequenced reads without trimming were mapped to the mouse reference genome sequences (mm10) using TopHat version 2.1.1 in combination with Bowtie 2 version 2.4.2. and SAMtools version 1.11. The fragments per kilobase of exon per million mapped fragments were calculated using Cufflinks version 2.2.1. Adjusted *P* values from RNA-sequencing data and principal component analysis (PCA) were analyzed by iDEP 9.1^44^. Upregulated genes were defined using a threshold of false discovery rate (FDR) < 0.1 and log2 fold change >1, following analysis with the DESeq2 package. Raw reads from these samples were submitted to the National Center for Biotechnology Information Gene Expression Omnibus database (accession number: GSE166982).

### Plasmids

Venus, Runx2, DN-Runx2, or Bmp2 cDNAs were ligated to the pAXcawt adenovirus vector (Takara) as described previously^45^. Flag-tagged-DN-Runx2 used in this study contains amino acids 2–247 of Runx2^11^. This construct lacks the transcriptional activation domain at the C-terminal region. The generation of these adenoviruses was performed using an adenovirus generation kit (Takara). A Venus adenovirus was used as the control adenovirus^46^. For shRNA vector construction for Smoc1 and Smoc2, the following oligo DNAs were used:

shSmoc1 forward, 5′-GATCCGCAAAGACTCCAAGTTGAATAATTCAAGAGATTATTCAACTTGGAGTCTTTGTTTTTTG-3′ and reverse, 5′-AATTCAAAAAACAAAGACTCCAAGTTGAATAATCTCTTGAATTATTCAACTTGGAGTCTTTGCG-3′; shSmoc2 forward, 5′-GATCCGCCAAGAATGACAATGTAGTGATTCAAGAGATCACTACATTGTCATTCTTGGTTTTTTG-3′ and reverse, 5′-AATTCAAAAAACCAAGAATGACAATGTAGTGATCTCTTGAATCACTACATTGTCATTCTTGGCG-3′; shGFP control forward, 5′-GATCCGCACAAGCTGGAGTACAACTACTTCAAGAGAGTAGTTGTACTCCAGCTTGTGTTTTTTG-3′ and reverse, 5′-AATTCAAAAAACACAAGCTGGAGTACAACTACTCTCTTGAAGTAGTTGTACTCCAGCTTGTGCG-3′. Each pair of single-stranded oligo DNAs was annealed at a concentration of 25 μM, and incubated at 95 °C for 5 min. The annealed oligo DNAs were individually inserted into the pSIREN-retroQ-based shRNA expression vector (Takara) at the BamHI/EcoRI site.

### Retrovirus infection

PLAT-E cells were seeded at 8 × 10^4^ cells/cm^2^ 1 day before transfection. Polyethylenimine (PEI) was used for all transfections. The pSIREN-retroQ shRNA expression vectors for shSmoc1 and shSmoc2 were mixed with PEI, and the plasmid-PEI complexes were incubated in Opti-MEM (Thermo Fisher) for 15 min at room temperature and added to PLAT-E cells. The virus supernatant was collected at 48 h after transfection and used to infect osteoblasts for 48 h in the presence of 4 μg/mL polybrene.

### Determination of ALP activity

ALP activity was determined as described previously^43,47,48^. In brief, cells were washed with PBS and solubilized with 0.1% Triton X-100, followed by determination of the ALP activity in lysates using *p*-nitrophenol phosphate as a substrate. Protein contents of the lysates were determined using the Bradford protein assay reagent (Bio-Rad, Hercules, CA, USA). For cytochemical analysis, cells were washed with PBS and fixed with 4% paraformaldehyde in PBS. Subsequently, cells were stained with a mixture of 330 μg/mL nitroblue tetrazolium, 175 μg/mL bromochloroindolyl phosphate, 100 mM NaCl, 50 mM MgCl_2_, and 100 mM Tris (pH 9.5).

### Alizarin red staining

Following induction of differentiation, cultured osteoblasts were washed with PBS twice, fixed in 70% ethanol, and stained with 0.4% alizarin red solution for 10 min.

### Skeletal preparation of mice

Following removal of the skin and viscera, mice were fixed in 96% ethanol for 24 h. Cartilage was stained for 24 h with alcian blue solution containing 0.015% alcian blue 8GX, 20% acetic acid, and 80% ethanol. Following dehydration with 100% ethanol for 3 days, the whole bodies were digested with 1% KOH at room temperature until the skeleton became clearly visible. The specimens were subsequently stained with 0.002% alizarin red in H_2_O for 24 h. Finally, the specimens were maintained in 100% glycerol and observed and photographed under an S-APO microscope (Leica, Wetzlar, Germany).

### Reverse transcription-quantitative polymerase chain reaction (RT-qPCR)

Cultured cells were washed twice with PBS and subjected to total RNA extraction with a Nucleo Spin RNA Plus Kit (Takara). cDNA was synthesized using ReverTra Ace^®^ qPCR RT Master Mix with gDNA Remover (TOYOBO, Osaka, Japan). The individual cDNAs were amplified with THUNDERBIRD^®^ SYBR qPCR Mix (TOYOBO) using a StepOnePlus™ Real-Time PCR System (Applied Biosystems, Foster City, CA, USA). The relative expression levels of the target genes were determined by the delta-delta Ct method using transcripts of *Actb* as the internal reference for each mouse RNA sample. The primer pairs and probes used for amplification were:

*Smoc1* forward, 5′-TGCCTGGGTGTTAGCAAAGAAG-3′, reverse, 5′-GCGTCCGATGAACGGGTTTG-3′, and probe, 5′-TGGTAGCCTTGGCAGCTTCCCTCAGG-3′; *Smoc2* forward, 5′-GCTTGGGTGTCACCAGAGAG-3′, reverse, 5′-CTGGGCTGTCTATTAGAAGAAGAAC-3′, and probe, 5′-AAGCCAACACCAGGAAGCGCCACA-3′; *Osterix* forward, 5′-AGCGACCACTTGAGCAAACAT-3′, reverse, 5′-GCGGCTGATTGGCTTCTTCT-3′, and probe, 5′-CCCGACGCTGCGACCCTCC-3′; *Osteocalcin* forward, 5′-GCAATAAGGTAGTGAACAGACTCC-3′, reverse, 5′-GTTTGTAGGCGGTCTTCAAGC-3′, and probe, 5′-TGGAGCCTCAGTCCCCAGCCCA-3′; *Runx2* forward, 5′-CTCCTTCCAGGATGGTCCCA-3′, reverse, 5′-CTTCCGTCAGCGTCAACACC-3′, and probe, 5′-CACCACCTCGAATGGCAGCACGCT-3′; *Actb* forward, 5′-TTAATTTCTGAATGGCCCAGGTCT-3′, reverse, 5′-ATTGGTCTCAAGTCAGTGTACAGG-3′, and probe, 5′-CCTGGCTGCCTCAACACCTCAACCC-3′.

### Whole-mount in situ hybridization

Digoxigenin (DIG)-labeled single-stranded RNA probes were prepared using a DIG RNA Labeling Kit (Roche, Basel, Switzerland). The *Smoc1* probe was an 847-bp fragment of the coding sequence (position 386–1232 in the NM_001146217.1 cDNA sequence). The *Smoc2* probe was a 1041-bp fragment of the coding sequence (position 460–1500 in the NM_022315.2 cDNA sequence). The *Runx2* probe was a 639-bp fragment of the coding sequence (position 3173–3807 in the NM_001146038.2 cDNA sequence).

C57BL6/J mouse embryos (E12.5) were fixed with 4% paraformaldehyde in PBS containing 0.1% Tween-20 overnight at 4 °C. Samples were hybridized with gene-specific DIG-labeled RNA probes overnight at 70 °C, washed, and incubated with 1:2500 diluted anti-DIG-AP Fab fragments (Roche) for 3 h at room temperature. For optimum signal detection, samples were treated with nitroblue tetrazolium chloride/5-bromo-4-chloro-3-indolyl phosphate at various times.

### Hematoxylin and eosin (HE) and von Kossa staining

Tissue preparation was conducted as previously described^49^. Briefly, mice tibiae at E15.5 were fixed in 4% paraformaldehyde in PBS and used to prepare paraffin-embedded sections with 7-µm thickness. The sections were deparaffinized and stained with Mayer’s hematoxylin and eosin solution. Bone mineralization was analyzed using the von Kossa staining method. In brief, the sections were fixed in 4% paraformaldehyde in PBS and subsequently incubated with 5% silver nitrate for 60 min under UV light. Following rinsing with distilled water, bone nodules were photographed using a phase-contrast microscope.

### In situ hybridization

Mice tibiae at E15.5 were fixed in 4% paraformaldehyde in PBS and used to prepare paraffin-embedded sections with 7-μm thickness. DIG-labeled single-stranded RNA probes were prepared using a DIG RNA Labeling Kit (Roche). The *Col2a1* probe was a 435-bp fragment of the coding sequence (position 1566–2000 in the NM_031163 cDNA sequence). The *Col10a1* probe was a 435-bp fragment of the coding sequence (position 903–1419 in the NM_009925.4 cDNA sequence). The *Col1a1* probe was a 324-bp fragment of the coding sequence (position 4466–4789 in the NM_007742.4 cDNA sequence). The *Mmp13* probe was a 639-bp fragment of the coding sequence (position 1576–2214 in the NM_008607.2 cDNA sequence). The *Pth1r* probe was a 780-bp fragment of the coding sequence (position 766–1545 in the XM_017313215.1 cDNA sequence). The *Osterix* probe was a 1312-bp fragment of the coding sequence (position 36–1347 in the NM_130458.3 cDNA sequence). The sections were mounted on glass slides, successively treated with 10 µg/mL proteinase K and 0.2 M HCl, and subjected to acetylation in 0.1 M triethanolamine/0.25% acetic anhydride. Following prehybridization, the sections were incubated with DIG-labeled RNA probes at 65 °C for 16 h. The sections were further incubated with anti-DIG-AP Fab fragments for 2 h at room temperature. Following washing, the sections were treated with nitroblue tetrazolium chloride/5-bromo-4-chloro-3-indolyl phosphate for various times for optimum staining.

### Generation of *Smoc1* flox mice and *Smoc2* KO mice

Three splice variants are known to exist for *Smoc1* at the protein level according to the Ensemble database (http://www.ensembl.org/index.html). All splice variants contain exon 1 (chromosome 12: 81026808–81027158), exon 2 (chromosome 12: 81104607–81104772), exon 3 (chromosome 12: 81105899–81106011), exon 4 (chromosome 12: 81135776–81135875), exon 5 (chromosome 12: 81137965–81138012), exon 6 (chromosome 12: 81150619–81150708), exon 7 (chromosome 12: 81152673–81152753), exon 8 (chromosome 12: 81167507–81167699), exon 9 (chromosome 12: 81168229–81168311), exon 10 (chromosome 12: 81170186–81170291), and exon 11 (chromosome 12: 81179479–81179723). Exon 1 includes the ATG start codon of *Smoc1*. Therefore, we decided to generate mutant mice, in which exon 1 of the *Smoc1* locus was floxed (Supplementary Fig. 6a). Similarly, three splice variants are known to exist for *Smoc2* at the protein level. All splice variants contain exon 1 (chromosome 17: 14279506–14279799), exon 2 (chromosome 17: 14325535–14325706), and exon 3 (chromosome 17: 14336547–14336653). Exon 1 includes the ATG start codon of *Smoc2*. Therefore, we decided to generate mutant mice, in which exon 1 of the *Smoc2* locus was flanked by the PGK-Neo cassette (Supplementary Fig. 7a). These targeting vectors were electroporated into TT2 embryonic stem cells^50^ and their homologous recombination was examined by Southern blotting analysis. Germline transmission of the mutant allele of *Smoc1* flox or *Smoc2* mutant was achieved by mating with C57BL/6J mice and confirmed by Southern blotting, genomic PCR, and RT-qPCR (Supplementary Materials and Methods, Supplementary Figs. 6b–d and 7b–d). The accession numbers for the *Smoc1* floxed and *Smoc2* heterozygous deficient mice are CDB0719K and CDB0802K, respectively (http://www2.clst.riken.jp/arg/mutant%20mice%20list.html). CAG-Cre transgenic mice were provided by the RIKEN Bioresource Research Center (Tsukuba, Japan; RBRC01828). For genotyping, the product sizes were: *Smoc1* WT) allele, 202 bp (forward primer, 5′-TCTCTCCCATTGGCTTCCAC-3′; reverse primer, 5′-GAGTGCGAGCGTGTGCTCT-3′); *Smoc1* deletion allele, 112 bp (forward primer, 5′-AACCGCCCCTCTCATCTCT-3′; reverse primer, 5′-GGTCCAGCGACACAACTTTAT-3′); *Smoc2* WT allele, 216 bp (forward primer, 5′-GTTCGCACACCGGATCTTC-3′; reverse primer, 5′-GGTTCTCAGTGAGGGACAACAG-3′); *Smoc2* KO allele, 205 bp (forward primer, 5′-GTTCGCACACCGGATCTTC-3′; reverse primer, 5′-GGTTCTCAGTGAGGGACAACAG-3′).

All protocols for animal use and experiments were approved by the Osaka University Institute Animal Experiment Committee and the Institutional Animal Care and Use Committee of the RIKEN Kobe Branch.

### Statistics and reproducibility

The statistical significances of differences in data were determined by two-tailed and unpaired Student’s *t* tests for two groups. Differences between three or more groups were compared by one-way analysis of variance (ANOVA) or two-way ANOVA followed by Tukey’s multiple-comparisons test. Values of *P* < 0.05 were considered to indicate statistical significance. All experiments were independently repeated at least three times under exactly the same conditions.

### Reporting summary

Further information on research design is available in the Nature Research Reporting Summary linked to this article.

## Supplementary information

Transparent Peer Review File

Supplementary Information

Description of Additional Supplementary Files

Supplementary Data 1

Supplementary Data 2

Supplementary Data 3

Supplementary Data 4

Reporting Summary

## Data Availability

The source data for the graphs and charts in the main figures are available as Supplementary Data 1, and any remaining information can be obtained from the corresponding author upon reasonable request.

## References

[1] Karsenty G (1998). Transcriptional regulation of osteoblast differentiation during development. Front. Biosci..

[2] Long F (2011). Building strong bones: molecular regulation of the osteoblast lineage. Nat. Rev. Mol. Cell Biol..

[3] Kronenberg HM (2003). Developmental regulation of the growth plate. Nature.

[4] Long F, Ornitz DM (2013). Development of the endochondral skeleton. Cold Spring Harb. Perspect. Biol..

[5] Nakashima K (2002). The novel zinc finger-containing transcription factor osterix is required for osteoblast differentiation and bone formation. Cell.

[6] Komori T (1997). Targeted disruption of Cbfa1 results in a complete lack of bone formation owing to maturational arrest of osteoblasts. Cell.

[7] Akiyama H, Chaboissier MC, Martin JF, Schedl A, de Crombrugghe B (2002). The transcription factor Sox9 has essential roles in successive steps of the chondrocyte differentiation pathway and is required for expression of Sox5 and Sox6. Genes Dev..

[8] Otto F (1997). Cbfa1, a candidate gene for cleidocranial dysplasia syndrome, is essential for osteoblast differentiation and bone development. Cell.

[9] Mundlos S (1997). Mutations involving the transcription factor CBFA1 cause cleidocranial dysplasia. Cell.

[10] Ducy P, Zhang R, Geoffroy V, Ridall AL, Karsenty G (1997). Osf2/Cbfa1: a transcriptional activator of osteoblast differentiation. Cell.

[11] Nishimura R, Hata K, Harris SE, Ikeda F, Yoneda T (2002). Core-binding factor alpha 1 (Cbfa1) induces osteoblastic differentiation of C2C12 cells without interactions with Smad1 and Smad5. Bone.

[12] Komori T (2010). Regulation of bone development and extracellular matrix protein genes by RUNX2. Cell Tissue Res..

[13] Inada M (1999). Maturational disturbance of chondrocytes in Cbfa1-deficient mice. Dev. Dyn..

[14] Malaval L (2008). Bone sialoprotein plays a functional role in bone formation and osteoclastogenesis. J. Exp. Med.

[15] Rittling SR (1998). Mice lacking osteopontin show normal development and bone structure but display altered osteoclast formation in vitro. J. Bone Min. Res..

[16] Ducy P (1996). Increased bone formation in osteocalcin-deficient mice. Nature.

[17] Moriishi T (2020). Osteocalcin is necessary for the alignment of apatite crystallites, but not glucose metabolism, testosterone synthesis, or muscle mass. PLoS Genet.

[18] Komori, T. Functions of osteocalcin in bone, pancreas, testis, and muscle. *Int. J. Mol. Sci.* **21**, 10.3390/ijms21207513 (2020). 10.3390/ijms21207513 PMC7589887 33053789

[19] Matsubara T (2008). BMP2 regulates Osterix through Msx2 and Runx2 during osteoblast differentiation. J. Biol. Chem..

[20] Vannahme C, Gösling S, Paulsson M, Maurer P, Hartmann U (2003). Characterization of SMOC-2, a modular extracellular calcium-binding protein. Biochem. J..

[21] Vannahme C (2002). Characterization of SMOC-1, a novel modular calcium-binding protein in basement membranes. J. Biol. Chem..

[22] Jang WG (2012). BMP2 protein regulates osteocalcin expression via Runx2-mediated Atf6 gene transcription. J. Biol. Chem..

[23] Meyer MB, Benkusky NA, Pike JW (2014). The RUNX2 cistrome in osteoblasts: characterization, down-regulation following differentiation, and relationship to gene expression. J. Biol. Chem..

[24] Okada I (2011). SMOC1 is essential for ocular and limb development in humans and mice. Am. J. Hum. Genet.

[25] Rainger J (2011). Loss of the BMP antagonist, SMOC-1, causes Ophthalmo-acromelic (Waardenburg Anophthalmia) syndrome in humans and mice. PLoS Genet.

[26] Marchant TW (2017). Canine brachycephaly is associated with a retrotransposon-mediated missplicing of SMOC2. Curr. Biol..

[27] Papenbrock T, Visconti RP, Awgulewitsch A (2000). Loss of fibula in mice overexpressing Hoxc11. Mech. Dev..

[28] Motamed, K. SPARC (osteonectin/BM-40). *Int. J. Biochem. Cell. Biol.* **31**, 1363–1366 (1999). 10.1016/s1357-2725(99)00090-4 10641790

[29] Sage H, Johnson C, Bornstein P (1984). Characterization of a novel serum albumin-binding glycoprotein secreted by endothelial cells in culture. J. Biol. Chem..

[30] Termine JD (1981). Osteonectin, a bone-specific protein linking mineral to collagen. Cell.

[31] Jundt G, Berghäuser KH, Termine JD, Schulz A (1987). Osteonectin—a differentiation marker of bone cells. Cell Tissue Res..

[32] Gilmour DT (1998). Mice deficient for the secreted glycoprotein SPARC/osteonectin/BM40 develop normally but show severe age-onset cataract formation and disruption of the lens. EMBO J..

[33] Boskey AL, Moore DJ, Amling M, Canalis E, Delany AM (2003). Infrared analysis of the mineral and matrix in bones of osteonectin-null mice and their wildtype controls. J. Bone Min. Res..

[34] Abouzeid H (2011). Mutations in the SPARC-related modular calcium-binding protein 1 gene, SMOC1, cause Waardenburg anophthalmia syndrome. Am. J. Hum. Genet..

[35] Jamshidi J (2017). A novel mutation in SMOC1 and variable phenotypic expression in two patients with Waardenburg anophthalmia syndrome. Eur. J. Med. Genet.

[36] Brazil DP, Church RH, Surae S, Godson C, Martin F (2015). BMP signalling: agony and antagony in the family. Trends Cell Biol..

[37] Matsumoto Y (2010). Conditional ablation of the heparan sulfate-synthesizing enzyme Ext1 leads to dysregulation of bone morphogenic protein signaling and severe skeletal defects. J. Biol. Chem..

[38] Klemenčič M, Novinec M, Maier S, Hartmann U, Lenarčič B (2013). The heparin-binding activity of secreted modular calcium-binding protein 1 (SMOC-1) modulates its cell adhesion properties. PLoS ONE.

[39] Thomas JT, Canelos P, Luyten FP, Moos M (2009). Xenopus SMOC-1 Inhibits bone morphogenetic protein signaling downstream of receptor binding and is essential for postgastrulation development in Xenopus. J. Biol. Chem..

[40] Vuilleumier R (2010). Control of Dpp morphogen signalling by a secreted feedback regulator. Nat. Cell Biol..

[41] Thomas, J. T. et al. SMOC can act as both an antagonist and an expander of BMP signaling. *eLife* **6**, 10.7554/eLife.17935 (2017). 10.7554/eLife.17935 PMC5360445 28323621

[42] Ichida F (2004). Reciprocal roles of MSX2 in regulation of osteoblast and adipocyte differentiation. J. Biol. Chem..

[43] Takahata Y (2011). Osteoblastic γ-aminobutyric acid, type B receptors negatively regulate osteoblastogenesis toward disturbance of osteoclastogenesis mediated by receptor activator of nuclear factor κB ligand in mouse bone. J. Biol. Chem..

[44] Ge SX, Son EW, Yao R (2018). iDEP: an integrated web application for differential expression and pathway analysis of RNA-Seq data. BMC Bioinforma..

[45] Hata K (2013). Arid5b facilitates chondrogenesis by recruiting the histone demethylase Phf2 to Sox9-regulated genes. Nat. Commun..

[46] Takahata Y (2019). Sox4 is involved in osteoarthritic cartilage deterioration through induction of ADAMTS4 and ADAMTS5. FASEB J..

[47] Katagiri T (1994). Bone morphogenetic protein-2 converts the differentiation pathway of C2C12 myoblasts into the osteoblast lineage. J. Cell Biol..

[48] Takahata Y (2012). Positive regulation by γ-aminobutyric acid B receptor subunit-1 of chondrogenesis through acceleration of nuclear translocation of activating transcription factor-4. J. Biol. Chem..

[49] Yoshida M (2015). The transcription factor Foxc1 is necessary for Ihh-Gli2-regulated endochondral ossification. Nat. Commun..

[50] Yagi T (1993). A novel ES cell line, TT2, with high germline-differentiating potency. Anal. Biochem..

