# Supplementary material for: Smoc1 and Smoc2 regulate bone formation as downstream molecules of Runx2
Source: Commun Biol. 2021 Oct 19;4:1199. doi: 10.1038/s42003-021-02717-7 (PMC8526618; doi:10.1038/s42003-021-02717-7)
Supplement: Supplementary file 2 — Supplementary Information [file 42003_2021_2717_MOESM2_ESM.pdf]

Supplementary information

Supplementary materials and method

Primer sets used for genotyping

Smoc1 WT Forward; 5’-TCTCTCCCATTGGCTTCCAC-3’, Reverse; 5’-GAGTGCGAGCGTGTGCTCT-3’

Smoc1 Deletion Forward; 5’-AACCGCCCCTCTCATCTCT-3’, Reverse; 5’-GGTCCAGCGACACAACTTTAT-3’

Smoc2 WT Forward; 5’-GTTCGCACACCGGATCTTC-3’, Reverse; 5’-GGTTCTCAGTGAGGGACAACAG-3’

Smoc2 Deletion Forward; 5’-TAGGTCCCTCGAAGAGGTTCA-3’, Reverse; 5’-TTGTCCCAGAAGGAAGAGTGG-3’

ChIP-seq data analysis using Integrative Genomics Viewer (IGV)

For the analysis of Runx2 binding region in mouse whole genome, we used public ChIP-seq data (GEO41920). Visualized the data that Runx2 binding region of the Smoc1 and Smoc2 genome were analyzed by IGV version 2.9.2 (Broad Institute and the Regents of the University of California.)

Chromatin immunoprecipitation assay

Chromatin immunoprecipitation (ChIP) analysis was performed by using a ChIP-IT Express Magnetic Chromatin Immunoprecipitation Kit (Active Motif, Carlsbad, CA, USA). Mouse osteoblasts were treated chromatin fixation with 1% formaldehyde for 5 min and then sonication to acquire DNA fragments of 300-1000 bp. Sonicated chromatin

samples were immunoprecipitated with an anti-Runx2 antibody (Cell Signaling Technology) conjugated to protein-G magnetic beads. DNA was eluted from immunoprecipitated samples and then amplified Smoc1 promoter region and intron, Smoc2 promoter region and intron. Following primer sets were used for PCR.

Smoc1 promoter Forward; 5'-CAGGTTGTCCTCAGGGTTGT-3', Reverse; 5'-AACCATGTCAGGCTTTCCAG-3'

Smoc1 intron Forward; 5'-TGTGCTTTGCAGAGGTTGTC-3', Reverse; 5'-GAAATGGCTCAGCCTCTACG-3'

Smoc2 promoter Forward; 5'-GGGCAGAAACAGATGGTGAC-3', Reverse; 5'-CAGGGGGAATAAAACCCAGT-3'

Smoc2 intron Forward; 5'-TGGCCAGCATACTGACATTT-3', Reverse; 5'-GATGGGGTCAGTCTCAGCTC-3'

Cells and reagents

HEK293T cells and AAVpro 293T cell line were purchased from RIKEN Cell Bank (Ibaraki, Japan) and Takara (Shiga, Japan), respectively. Cells were cultured at 37 °C in a humidified 5% CO_2 incubator with DMEM (Sigma, Saint Louis, MO, USA) containing 10 % fetal bovine serum.

Immunoprecipitation and western blotting

Cells were washed twice with PBS and lysed in RIPA buffer [50 mM Tyrs-HCl (pH7.4), 150 mM NaCl, 1mM EDTA, 0.25% deoxycholic acid, and 1% NP-40] containing a protease inhibitor cocktail (Nacalai Tesque, Kyoto, Japan). Lysates were centrifuged for 5 min at 4°C at 20,000 g, and supernatants were incubated with the anti-PA antibody

(Wako, Japan) for 3 h at 4°C and subsequent immunoprecipitation for 3h with protein G magnetic beads (Thermo Fisher). Immunoprecipitants were washed three times with RIPA buffer and boiled in SDS sample buffer. Protein samples were separated by SDS-PAGE, followed by transfer to PVDF membranes. The membranes were incubated with anti-Flag (1:10000, 66008-3-Ig, Wako) or anti-PA (1:10000, 012-25863, Wako) antibody for 16 h at 4°C. Subsequently, membranes were incubated with anti-mouse (1:10000, 458; Medical and Biological Laboratories, Nagoya, Japan) or anti-Rat (1:10000, 7077; Cell Signalin Technology) IgGs conjugated with horseradish peroxidase for 1 h at room temperature. Finally, the membrane was incubated with an ECL detection reagent.

Plasmids and Adeno-associated virus (AAV)

ZsGreen coding sequence was used pZsGreen vector (Takara), and cDNA was subcloned into pcDNA3.1 expression vector. Codon optimized full-length Hoxc11 cDNA was synthesized and subcloned into pcDNA3.1 expression vector containing a 3xFlag at the N-terminal. Full-length Runx2 cDNA amplified by PCR were subcloned into pcDNA3.1 expression vector containing a PA-tag at the N-terminal. For the AAV production, Zsgreen and Hoxc11 cDNA was subcloned into pAAV-CMV vector (Takara), and generated by commercial kit using AAVpro Helper Free system (Takara).

Alcian Blue staining

Cells were fixed with 4% paraformaldehyde in PBS and stained with 1% Alcian Blue 8GX solution.

Southern blotting

DNA was extracted from mouse tail tissue and samples were digested with endonuclease. The samples were separated by agarose gel electrophoresis and transferred to nitrocellulose membranes. Membranes were hybridized with ^{32}P-labeled probes, washed, and exposed to X-ray film. The sequences of the probes are shown below.

Smoc1 probe sequence:

5’-

cattgcaccaggagaagctgcagagcaatctttcttatccatgttggtaaagaattatgcttggatttcaaccctgtctcaaaagat

accagcttccccagaaatgtggctctggtcaagagccacagggtcataggaagagcagaaacatctggcttgttgcagaattc

cctttgggcacccgaggtctcctctcttaatcttttgagtgacagaagcttaaggattgccatctttcccagctggctttctaggaa

accgagaacatgccctttcgctttaactatctgactcactaattgagctcccttggggcctagaccccaatccttgcccccttttctg

cttcctgcctcccttcataaaatttctgacatttgtttgactctggaaaccaccagctatcaaatatcagattggaggttgctctatag

gaagcacctgatgctccgaggtctaagcagccacttagctgtctgggaagctggtcagcatcc-3’

Smoc2 probe sequence:

5’-

acccagcatgtcctaagggttaaaggtaacatttgatgcactcagactacactcattctccaatccagattatgctgttggaaggg

agtttagaagccagctcttacactgttgtgtcattacggggtgtttgggtattagagtagattccttgcctggcttagatgagagaa

atcatgtgggctgtccaggcagatgggaagagcacgttcttccctttgtcctgcccatttgttctgggaatgagtaaactgattca

cctcttggggaggaagtgtgtacggacagagccagccgacagagccagcctgtggatttttccacccttctcatggcctggtt

gtgcacagatgttagcgttctttccagtgagtggagtgaggtgaggagatgcgggcattctgccgtgtgctttgtgattttcagct

tgggtctttgaagggaaggagagtgatgggaagacagcagtttggacacgtggattcagaagaacaccactgatgatcatca

gtccaggacaggacgt-3’

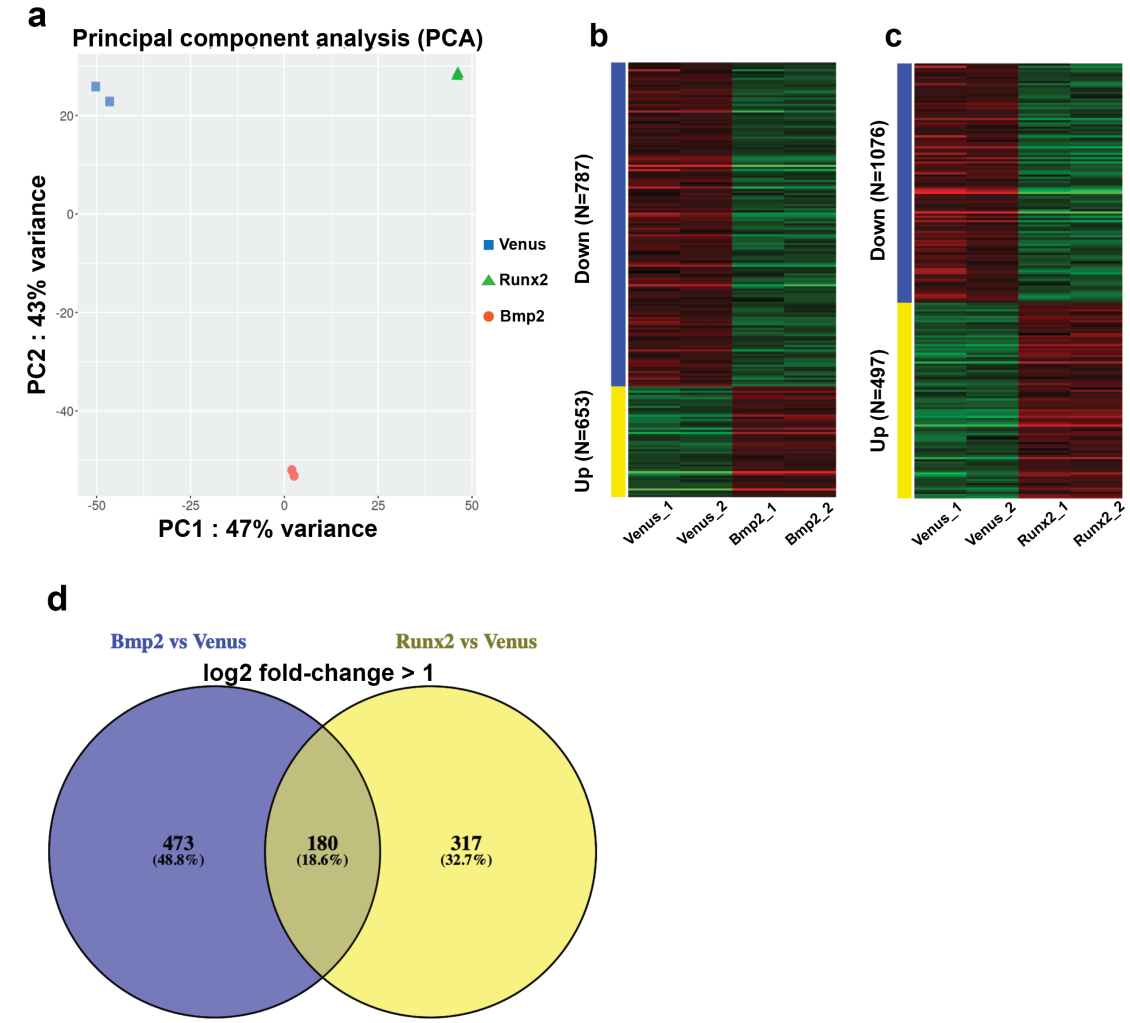

Supplementary Figure 1. Schematic diagram of genes upregulated or downregulated by Runx2 and Bmp2. Primary limb bud cells were infected with Venus (control), Runx2, or Bmp2 adenoviruses. At 4 days after infection, total RNA was extracted from the cells and RNA-sequence analyses were performed ($n = 2$, biologically independent samples). **(a)** Samples of each group were examined via principal component analysis. Genes with log2 fold-change values > 1 were considered upregulated, while genes with log2 fold-change values < -1 were considered downregulated, by Bmp2 (**b**) and Runx2 (**c**) compared to Venus control. (**d**) Venn diagram shows Bmp2 vs Venus and Runx2 vs Venus. The details of these genes are listed in the Supplementary Data 2 and 3.

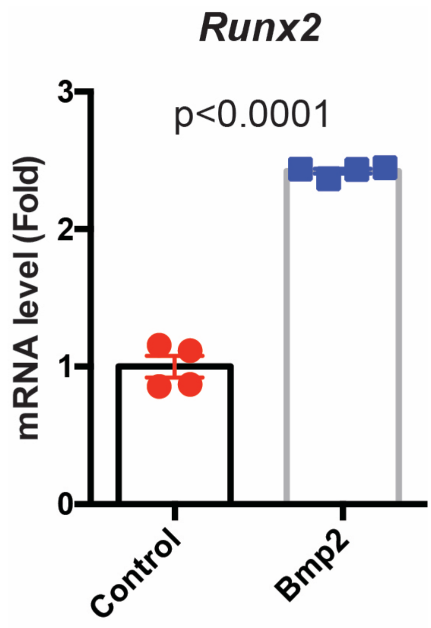

Supplementary Figure 2. Bmp2-induced *Runx2* expression in limb bud cells. Limb bud cells were incubated with or without Bmp2 (500 ng/mL) for 4 days. Total RNA was extracted from the cells and *Runx2* expression was determined by RT-qPCR. Values are the mean ± SE. (n = 4, biologically independent samples)

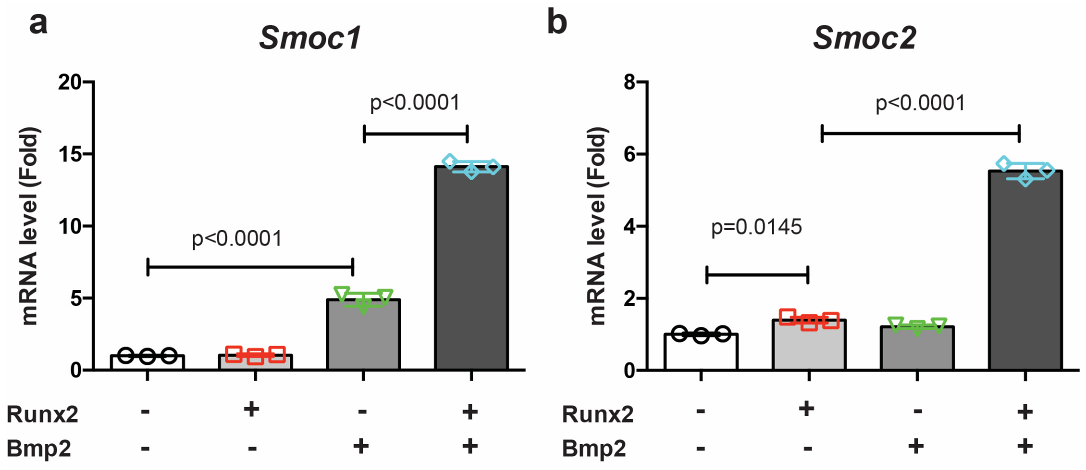

Supplementary Figure 3. Cooperative effects of Bmp2 and Runx2 on *Smoc1* and *Smoc2* expressions. (**a, b**) Total RNA was extracted from limb bud cells cultured for 24 h after infection with Runx2 and/or Bmp2 adenoviruses. Control cells were infected with the Venus adenovirus. Expression of *Smoc1* (**a**) and *Smoc2* (**b**) mRNAs in the cells was determined by RT-qPCR. Values are the mean ± SE. (n = 3, biologically independent samples).

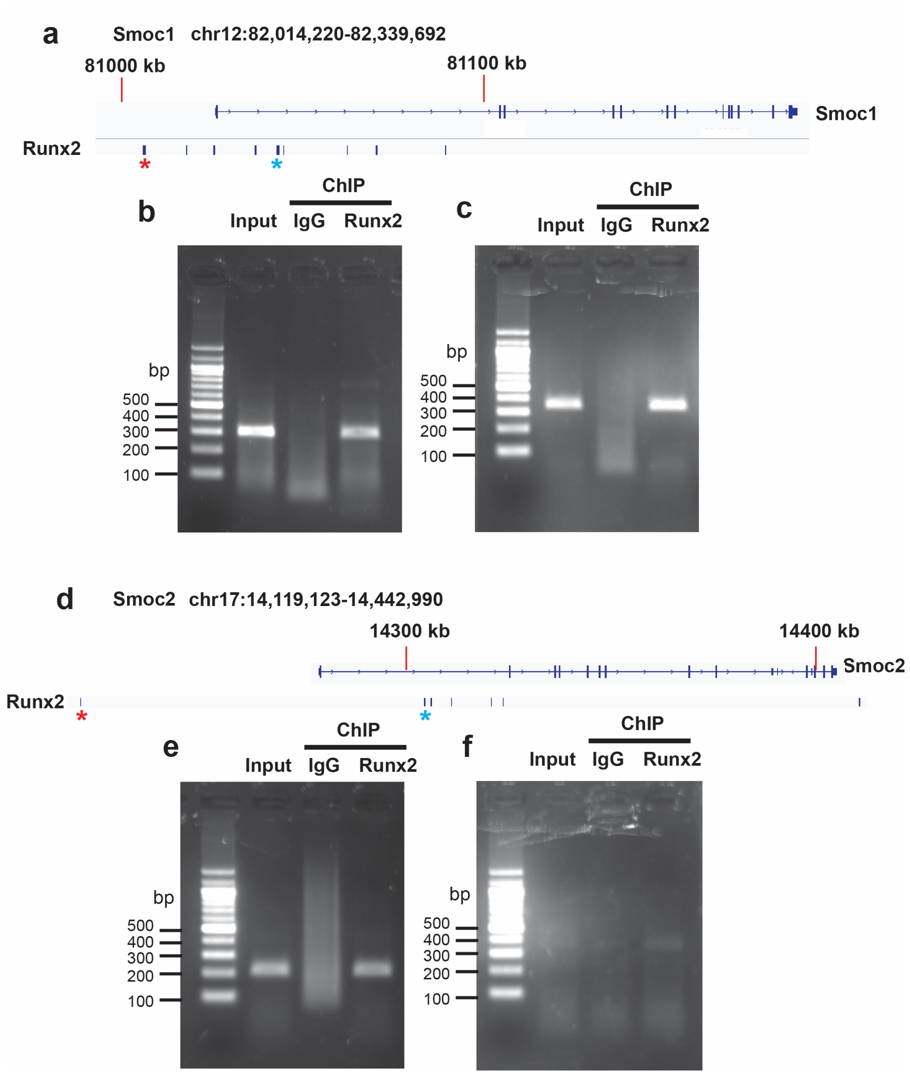

Supplementary Figure 4. Direct association of Runx2 with the *Smoc1* and *Smoc2* genes. Runx2 ChIP-seq datasets for osteoblastic MC3T3E1 cells were obtained from GSE41920. (**a**) The Runx2 ChIP-seq data of the *Smoc1* gene region were visualized on the IGV genome browser. (**b, c**) Mouse osteoblasts were subjected to ChIP analysis using control IgG or anti-Runx2 antibody. PCR amplification regions for this study are shown by a red asterisk (**b**) and a blue asterisk (**c**), as shown in (**a**), respectively. (**d**) The Runx2 ChIP-seq data of the *Smoc2* gene region were visualized on the IGV genome browser. (**e, f**) Mouse osteoblasts were subjected to ChIP analysis using control IgG or anti-Runx2 antibody. PCR amplification regions for this study are shown by a red asterisk (**e**) and a blue asterisk (**f**), as shown in (**d**), respectively.

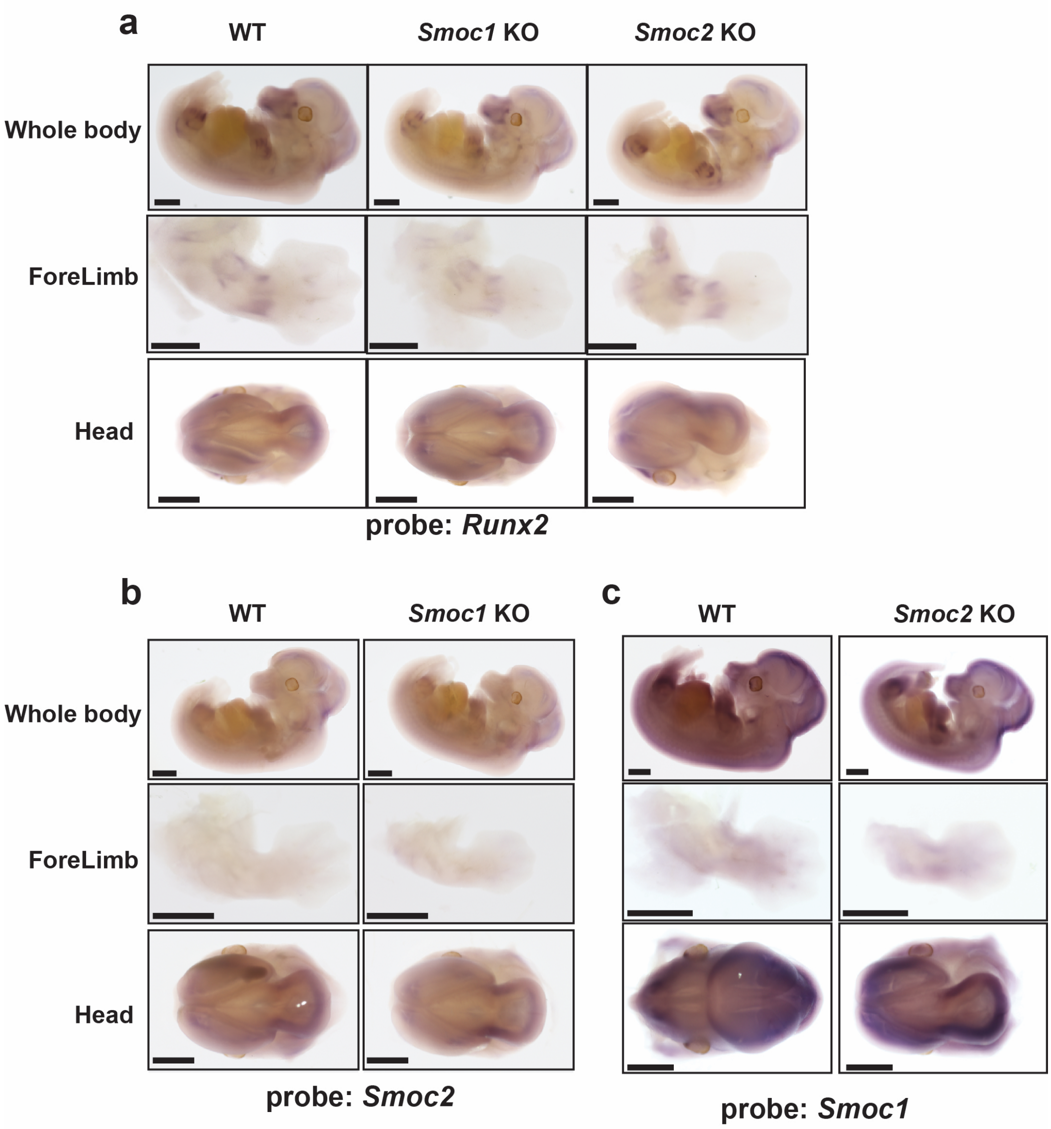

Supplementary Figure 5. Expression of *Runx2*, *Smoc1*, or *Smoc2* in WT, *Smoc1* KO, and *Smoc2* KO lines. (**a**) Whole-mount *in situ* hybridization analyses in WT, *Smoc1* KO, and *Smoc2* KO mice at E12.5 for *Runx2*. (**b**) Whole mount *in situ* hybridization analyses of WT and *Smoc1* KO mice at E12.5 for *Smoc2*. (**c**) Whole mount *in situ* hybridization analyses of WT and *Smoc2* KO mice at E12.5 for *Smoc1*. Scale bar: 1mm. (n = 3, biologically independent animals)

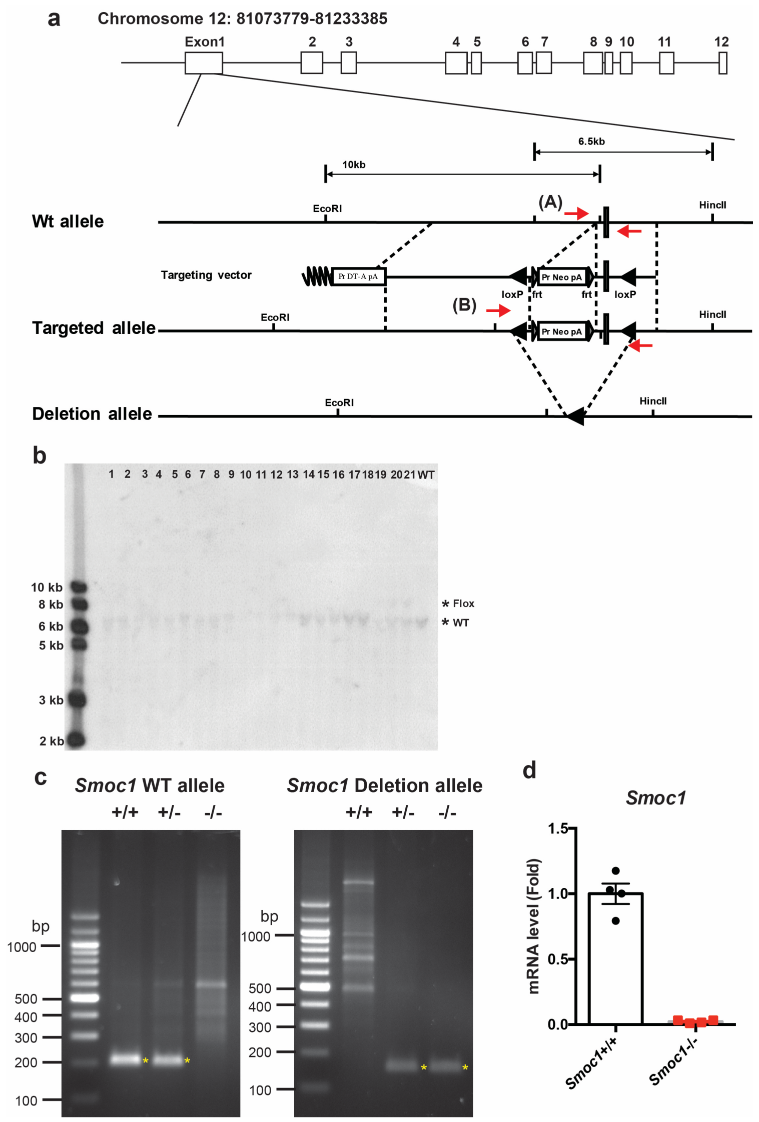

Supplementary Figure 6. Confirmation of the targeting of the Smoc1 genes in mutant mice. (**a**) Schematic diagram of the *Smoc1* flox allele. Arrows (A): genotyping primer pair for the WT allele; arrows (B): genotyping primer pair for the flox-deficient allele. (**b**) Genomic DNA was extracted from the tail tissue of each mouse. Southern blotting analysis was used to verify the Smoc1 floxed allele (WT 6.5 kb, floxed mutant 8 kb). (**c**) Genomic DNA was extracted from the tail of each indicated mouse genotype. PCR analysis verifying the wild-type and *Smoc1* deletion allele in *Smoc1* mutant mice. (+/+ WT, +/− heterozygous mutant, −/− homozygous mutant mice) (**d**) Total RNA was extracted from limb bud cells of WT control or *Smoc1* KO mice, and the *Smoc1* mRNA expression was determined by RT-qPCR. Values are the mean ± SE (WT; n = 4 KO; n = 4, biologically independent animals).

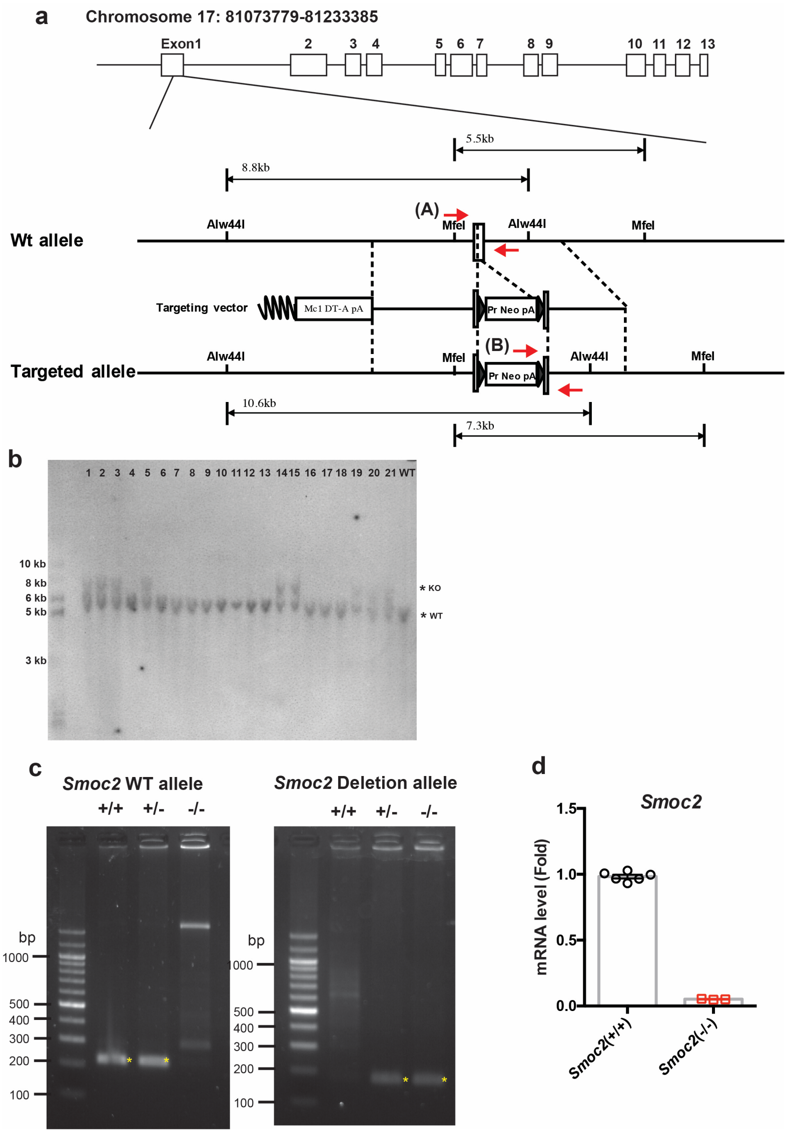

Supplementary Figure 7. Confirmation of the targeting of the Smoc2 genes in mutant mice. (**a**) Schematic diagram of the *Smoc2* mutant allele. Arrows (A): genotyping primer pair for the WT allele; arrows (B): genotyping primer pair for the deficient allele. (**b**) Genomic DNA was extracted from the tail tissue of each mouse. Southern blotting was used to verify the Smoc2 mutation allele (WT 5.5 kb, Mutant 7.3 kb). (**c**) Genomic DNA was extracted from the tail of each indicated mouse genotype. PCR analysis verifying the wild-type and deletion allele in *Smoc2* mutant mice. (+/+ WT, +/− heterozygous mutant, −/− homozygous mutant mice) (**d**) Total RNA was extracted from limb bud cells of WT control or *Smoc2* KO mice, and the *Smoc2* mRNA expression was determined by RT-qPCR. Values are the mean ± SE (WT; n = 6 KO; n = 3, biologically independent animals).

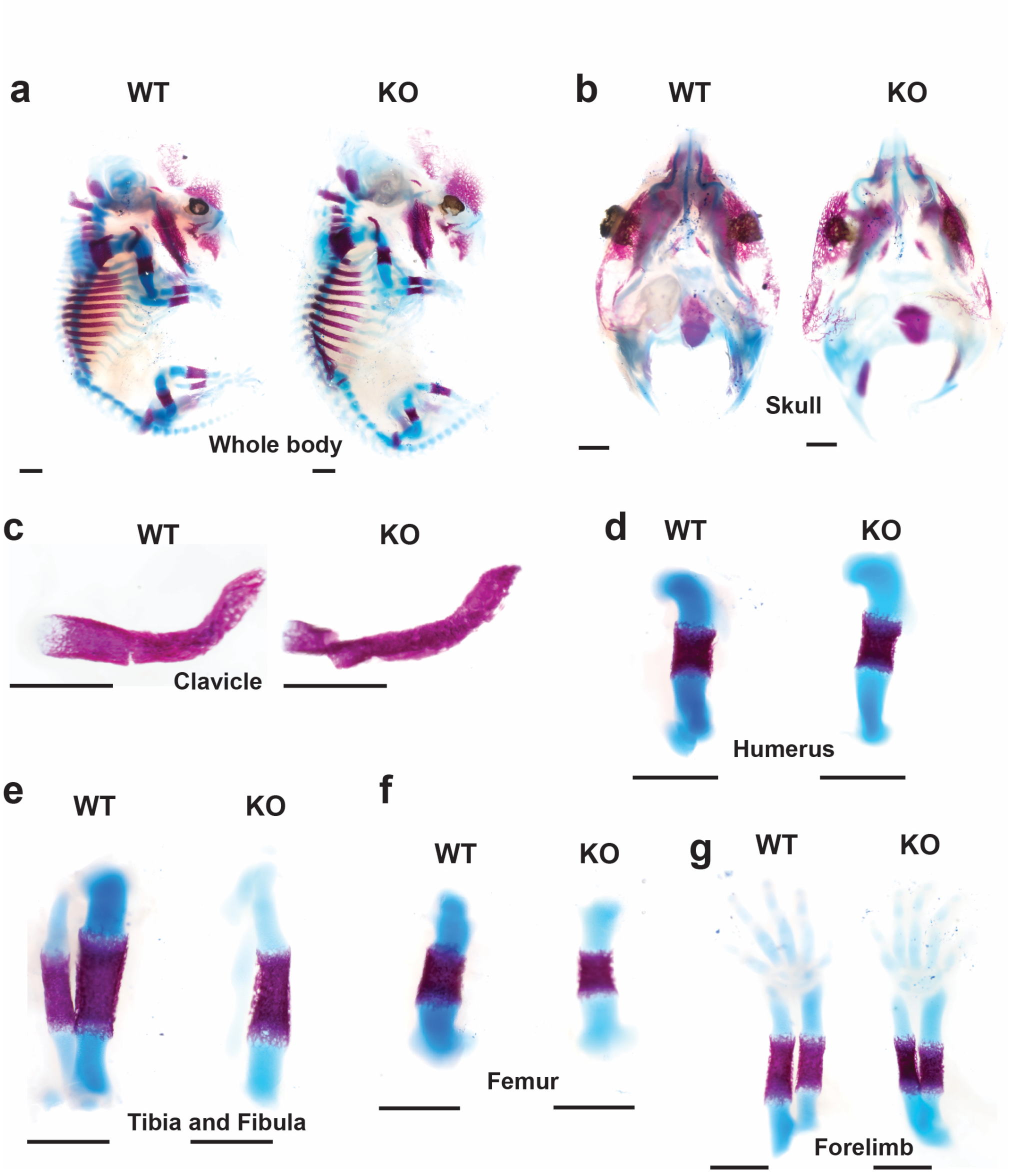

Supplementary Figure 8. Skeletal preparation of *Smoc1* KO mice. *Smoc1*$^{+/-}$ mice were mated, and *Smoc1* KO and WT littermate mice at E16.5 were subjected to alizarin red and alcian blue staining. Staining in the whole body (**a**), skull (dorsal view) (**b**), clavicle (**c**), humerus (**d**), tibia and fibula (**e**), femur (**f**), and forelimb (**g**) of *Smoc1* KO and WT littermate mice is shown. Scale bars: 0.5 mm. (n = 6, biologically independent animals)

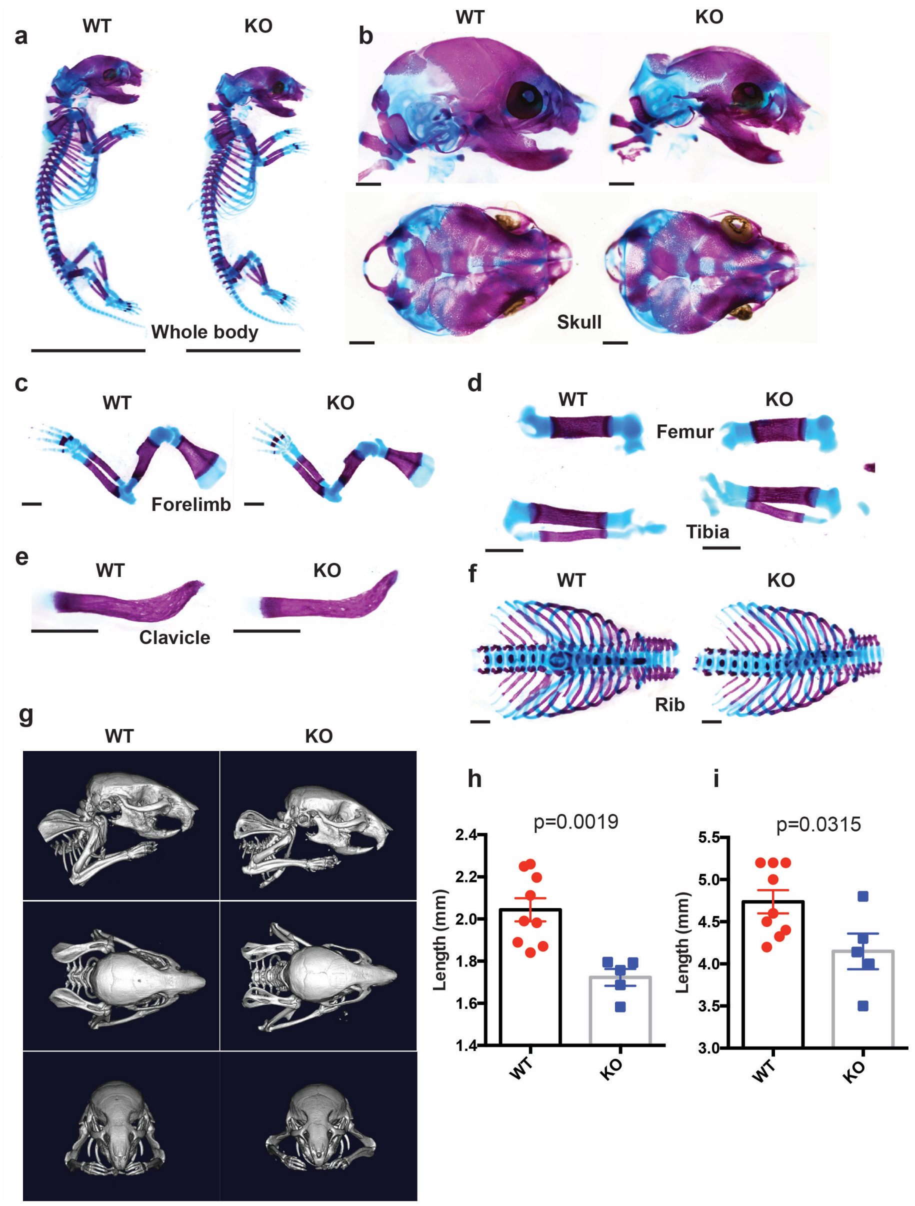

Supplementary Figure 9. Skeletal preparation of *Smoc2* KO mice. (**a–f**) *Smoc2*$^{+/-}$ mice were mated, and *Smoc2* KO and WT littermate mice at E18.5 were subjected to alizarin red and alcian blue staining. Staining in the whole body (**a**), skull (lateral and dorsal views) (**b**), forelimb (**c**), femur (upper) and tibia (lower) (**d**), clavicle (**e**), and ribs (**f**) of *Smoc2* KO and WT littermate mice is shown. Scale bar (**a**): 1 cm. Scale bars (**b–f**): 1 mm. (n = 10.) (**g**) Micro CT analysis of *Smoc2* KO and WT littermate mice at 8 weeks. (n = 4.) Distance from nasal bone to eyes (**h**) and nasal bones to parietal bone (**i**) in *Smoc2* KO and WT mice at the E18.5 stage. Values are the mean ± SE. (WT; n = 9, KO; n = 5, biologically independent animals)

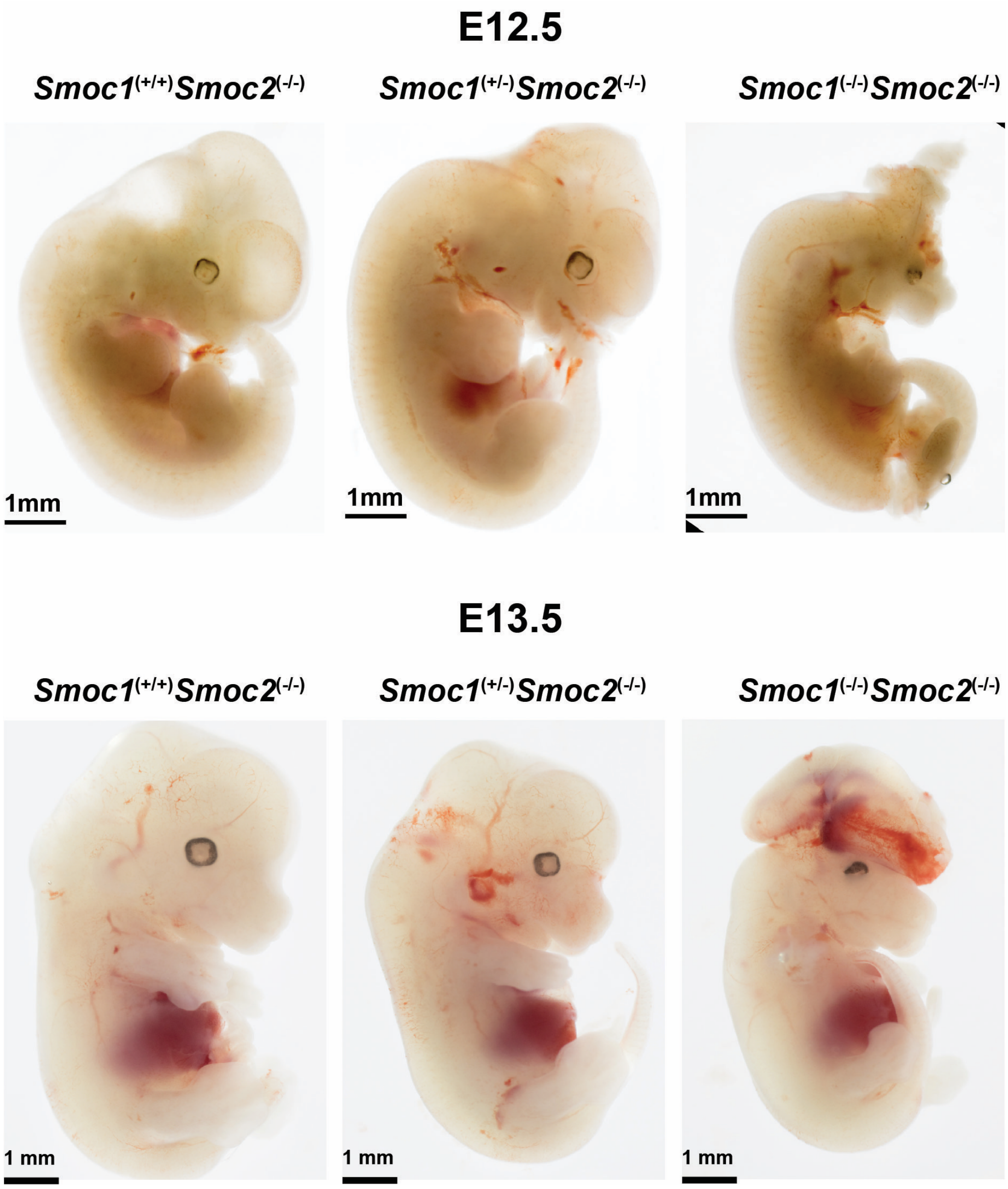

Supplementary Figure 10. Analysis of whole appearance of *Smoc1*;*Smoc2* mutant mice at E12.5 and E13.5. *Smoc1*$^{+/-}$;*Smoc2*$^{-/-}$ mice were mated, and the *Smoc1*$^{+/+}$;*Smoc2*$^{-/-}$, *Smoc1*$^{+/-}$;*Smoc2*$^{-/-}$, and *Smoc1*$^{-/-}$;*Smoc2*$^{-/-}$ littermate mice were macroscopically analyzed at E12.5 and E13.5 under a stereoscopic microscope.

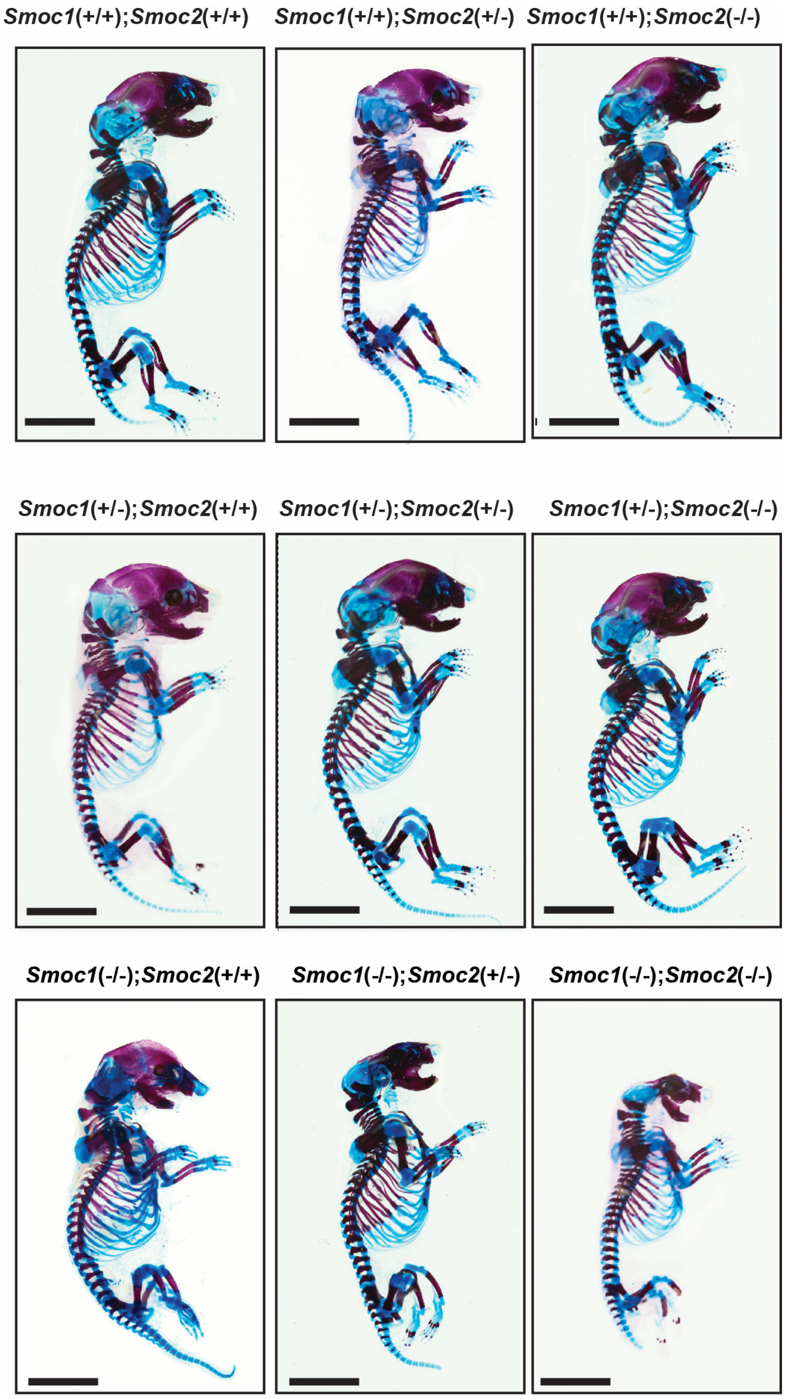

Supplementary Figure 11. Skeletal preparation of *Smoc1*;*Smoc2* mutant mice of all genotyping patterns. *Smoc1*$^{+/-}$;*Smoc2*$^{+/-}$ mice were mated, and all resulting genotype patterns were analyzed at E18.5 macroscopically under a stereoscopic microscope of skeletal preparations stained with alizarin red and alcian blue. Scale bars: 5 mm.

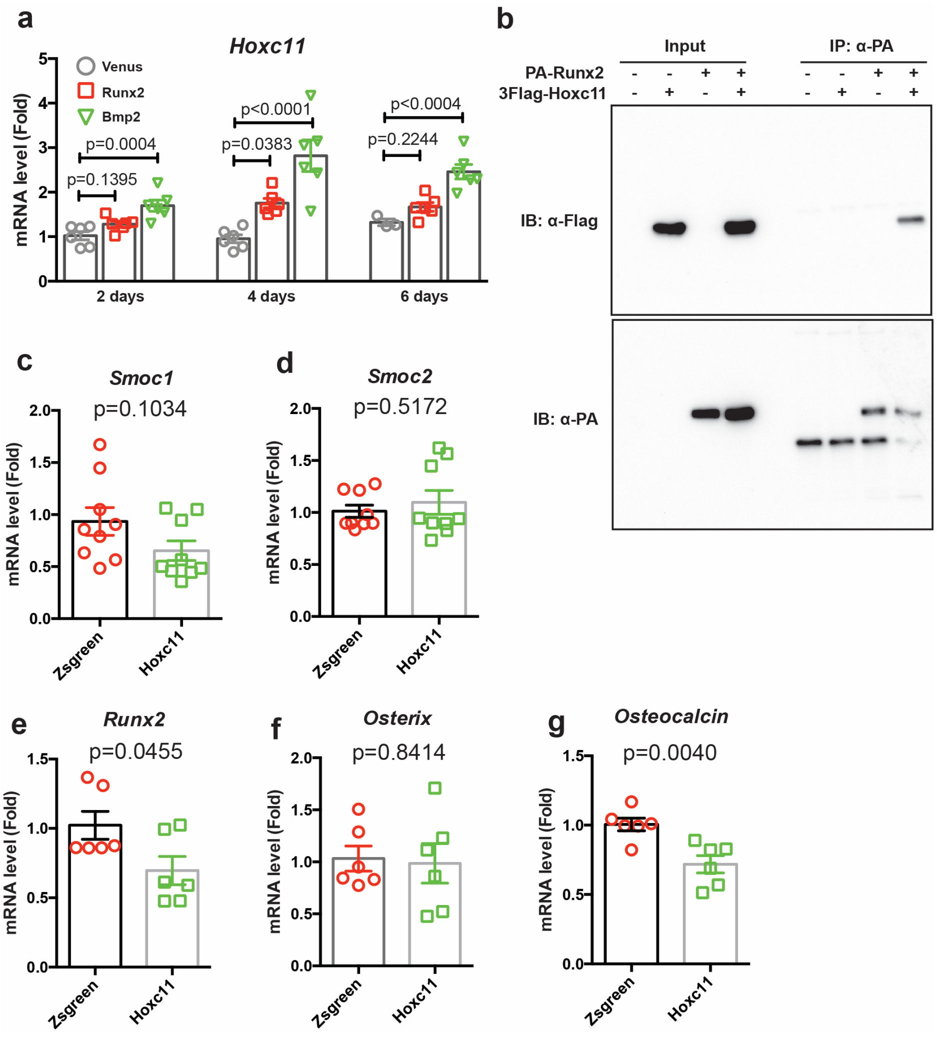

Supplementary Figure 12. Analysis of Hoxc11 function on *Smoc1* and *Smoc2* expressions. (**a**) Primary limb bud cells were infected with Venus (control), Runx2, or Bmp2 adenoviruses. At 2, 4, or 6 days after infection, total RNA was extracted from the cells and *Hoxc11* mRNA expression was determined by RT-qPCR. Values are mean ± SE (*n* = 6–10, biologically independent samples) (**b**) Lysates of 293T cells transfected with or without PA-Runx2 and/or 3Flag-Hocx11 were immunoprecipitated with anti-PA antibody, followed by determination of physical interaction by western blot with anti-Flag antibody. IP: immunoprecipitation. (**c–g**) Limb bud cells were infected with the adeno-associated virus of Zsgreen (control) or Hoxc11, and the mRNA expression of (**c**) *Smoc1*, (**d**) *Smoc2*, (**e**) *Runx2*, (**f**) *Osterix*, and (**g**) *Osteocalcin* by RT-qPCR was determined. Values are the mean ± SE. (*n* = 6–12, biologically independent samples)

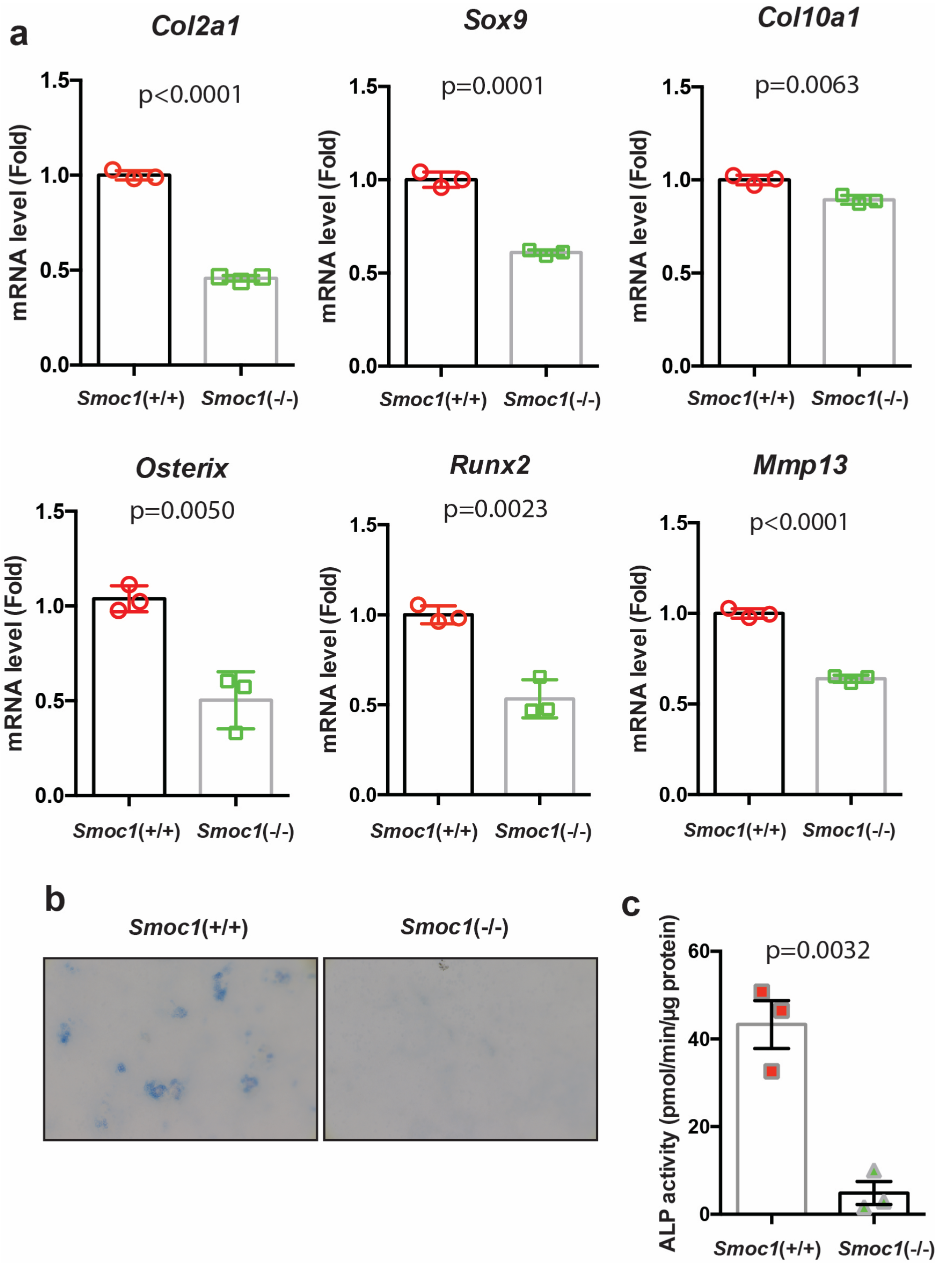

Supplementary Figure 13. Role of Smoc1 in osteoblast and chondrocyte differentiation. (**a**) Mouse limb bud cells were prepared from *Smoc1*$^{+/+}$ or *Smoc1*$^{-/-}$ mice, and cultured in the presence of 500 ng/mL Bmp2 and 50 μg/mL ascorbic acid. After 7 days, *Col2a1*, *Sox9*, *Col10a1*, *Osterix*, *Runx2*, and *Mmp13* mRNA expressions were determined by RT-qPCR. Values are the mean ± SE. (n = 3, biologically independent samples). The cells were evaluated by (**b**) alcian blue staining and (**c**) alkaline phosphatase activity, respectively (n = 3, independent experiments).

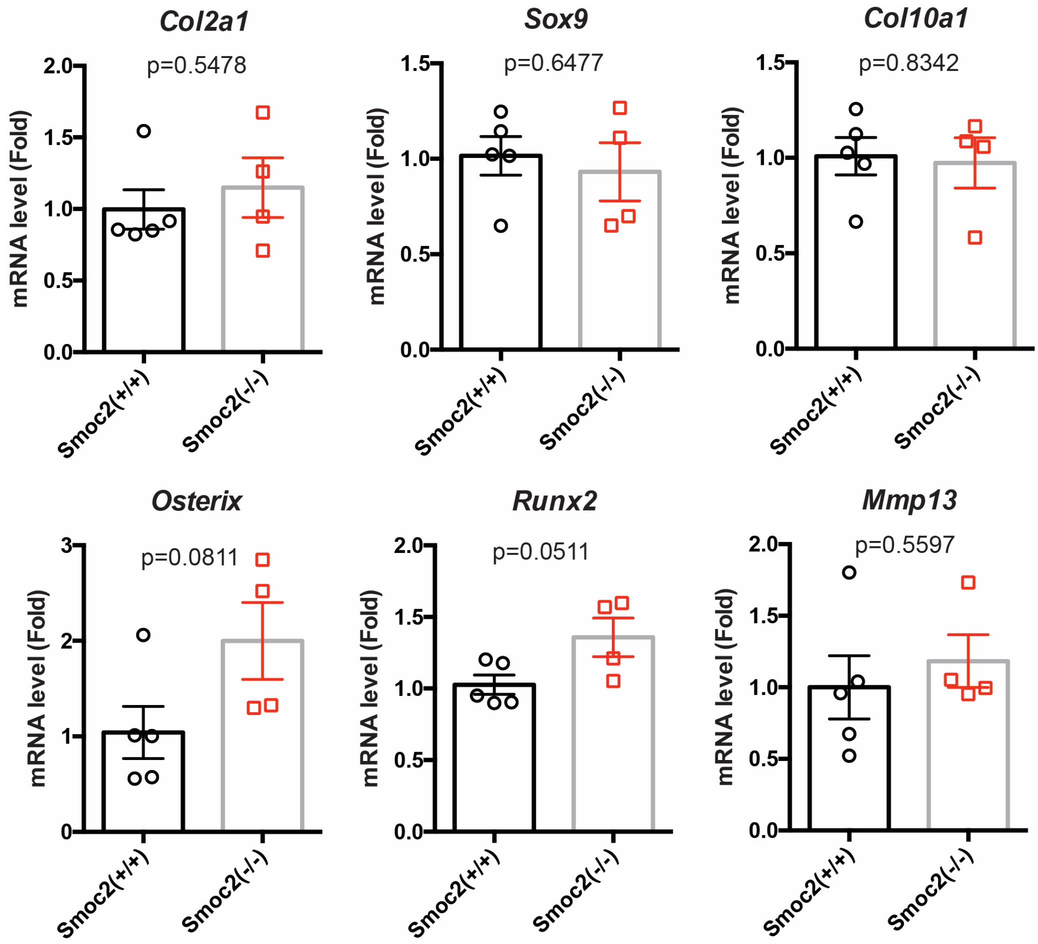

Supplementary Figure 14. Role of Smoc2 in chondrocyte differentiation. (**a–f**) Mouse limb bud cells were prepared from *Smoc2*$^{+/+}$ or *Smoc2*$^{-/-}$ mice, and cultured in the presence of 500 ng/mL Bmp2 and 50 µg/mL ascorbic acid. After 7 days, mRNA expressions of (**a**) *Col2a1*, (**b**) *Sox9*, (**c**) *Col10a1*, (**d**) *Osterix*, (**e**) *Runx2*, and (**f**) *Mmp13* were determined by RT-qPCR. Values are the mean ± SE. (*Smoc2* (+/+); *n* = 5, *Smoc2* (−/−); *n* = 4, biologically independent animals).
