# Supplementary material for: Smoc1 and Smoc2 regulate bone formation as downstream molecules of Runx2
Source: Commun Biol. 2021 Oct 19;4:1199. doi: 10.1038/s42003-021-02717-7 (PMC8526618; doi:10.1038/s42003-021-02717-7)
Supplement: Supplementary file 3 — Description of Additional Supplementary Files [file 42003_2021_2717_MOESM3_ESM.pdf]

Description of Additional Supplementary Files

File name: Supplementary Data 1

Description: (Smoc1(+/-); Smoc2(+/-) x Smoc1(+/-); Smoc2(+/-) E18.5 stage).

File name: Supplementary Data 2

Description: Differential gene expression in limb bud cells. Bmp2 vs Venus control

File name: Supplementary Data 3

Description: Differential gene expression in limb bud cells. Runx2 vs Venus control.

File name: Supplementary Data 4

Description: Source data for main figures.
