# Supplementary material for: Smoc1 and Smoc2 regulate bone formation as downstream molecules of Runx2
Source: Commun Biol. 2021 Oct 19;4:1199. doi: 10.1038/s42003-021-02717-7 (PMC8526618; doi:10.1038/s42003-021-02717-7)
Supplement: Supplementary file 8 — Reporting Summary [file 42003_2021_2717_MOESM8_ESM.pdf]

nature portfolio

Corresponding author(s): Yoshifumi Takahata

Last updated by author(s): Aug 23, 2021

Reporting Summary

Nature Portfolio wishes to improve the reproducibility of the work that we publish. This form provides structure for consistency and transparency in reporting. For further information on Nature Portfolio policies, see our Editorial Policies and the Editorial Policy Checklist.

Statistics

For all statistical analyses, confirm that the following items are present in the figure legend, table legend, main text, or Methods section.

n/a	Confirmed	
☐	☒	The exact sample size (*n*) for each experimental group/condition, given as a discrete number and unit of measurement
☐	☒	A statement on whether measurements were taken from distinct samples or whether the same sample was measured repeatedly
☐	☒	The statistical test(s) used AND whether they are one- or two-sided<br>*Only common tests should be described solely by name; describe more complex techniques in the Methods section.*
☐	☒	A description of all covariates tested
☐	☒	A description of any assumptions or corrections, such as tests of normality and adjustment for multiple comparisons
☐	☒	A full description of the statistical parameters including central tendency (e.g. means) or other basic estimates (e.g. regression coefficient) AND variation (e.g. standard deviation) or associated estimates of uncertainty (e.g. confidence intervals)
☐	☒	For null hypothesis testing, the test statistic (e.g. *F*, *t*, *r*) with confidence intervals, effect sizes, degrees of freedom and *P* value noted<br>*Give P values as exact values whenever suitable.*
☒	☐	For Bayesian analysis, information on the choice of priors and Markov chain Monte Carlo settings
☒	☐	For hierarchical and complex designs, identification of the appropriate level for tests and full reporting of outcomes
☒	☐	Estimates of effect sizes (e.g. Cohen's *d*, Pearson's *r*), indicating how they were calculated

Our web collection on statistics for biologists contains articles on many of the points above.

Software and code

Policy information about availability of computer code

Data collection	RNA-seq analysis was performed by using iDEP9.1 software
Data analysis	Statistic analysis was performed by using Prism6 software.

For manuscripts utilizing custom algorithms or software that are central to the research but not yet described in published literature, software must be made available to editors and reviewers. We strongly encourage code deposition in a community repository (e.g. GitHub). See the Nature Portfolio guidelines for submitting code & software for further information.

Data

Policy information about availability of data

All manuscripts must include a data availability statement. This statement should provide the following information, where applicable:

- Accession codes, unique identifiers, or web links for publicly available datasets
- A description of any restrictions on data availability
- For clinical datasets or third party data, please ensure that the statement adheres to our policy

RNA-seq datasets were submitted to the National Center for Biotechnology Information Gene Expression Omnibus database (accession number: GSE166982).

Field-specific reporting

Life sciences study design

All studies must disclose on these points even when the disclosure is negative.

Sample size	The sample size was usually within the range of the amount of experiment that one person could perform at lease three times independently, and determined that could be statistically analyzed.
Data exclusions	In the analysis of qRT-PCR, when RNA sample could not be prepared from cells due to technical failure, it is excluded from the data.
Replication	At least three times independent experiments were performed and reproducibility was confirmed.
Randomization	Mouse samples were taken from the same fetal period and grouped by determined genotype PCR.
Blinding	Investigators were not blinded.

Reporting for specific materials, systems and methods

We require information from authors about some types of materials, experimental systems and methods used in many studies. Here, indicate whether each material, system or method listed is relevant to your study. If you are not sure if a list item applies to your research, read the appropriate section before selecting a response.

Materials & experimental systems

n/a	Involved in the study	
☐	☒	Antibodies
☐	☒	Eukaryotic cell lines
☒	☐	Palaeontology and archaeology
☐	☒	Animals and other organisms
☒	☐	Human research participants
☒	☐	Clinical data
☒	☐	Dual use research of concern

Methods

n/a	Involved in the study	
☒	☐	ChIP-seq
☒	☐	Flow cytometry
☒	☐	MRI-based neuroimaging

Antibodies

Antibodies used	Anti DYKDDDDK tag, Monoclonal Antibody, code: 014-22383, Clone No: 1E6 , WAKO<br>Anti PA tag, Rat Monoclonal Antibody, code: 012-25863, Clone No: NZ01, WAKO
Validation	Data provided in the Supplementary information.

Eukaryotic cell lines

Policy information about cell lines

Cell line source(s)	LentiX-293T, Plat-E
Authentication	We did not perform Cell Line Authentication, but freshly used cells bought from supplier Takara bio corp.
Mycoplasma contamination	Cells lines were not tested for mycoplasma contamination
Commonly misidentified lines (See ICLAC register)	N/A

Animals and other organisms

Policy information about studies involving animals; ARRIVE guidelines recommended for reporting animal research

Laboratory animals	Mouse, C57BL6/J, Japan SLC,Inc
Wild animals	N/A
Field-collected samples	N/A
Ethics oversight	All protocols for the animal use and experiments were approved by the Osaka University Institute Animal Experiment Committee and the Institutional Animal Care and Use Committee of the RIKEN Kobe Branch

Note that full information on the approval of the study protocol must also be provided in the manuscript.
