# Supplementary material for: Smoc1 and Smoc2 regulate bone formation as downstream molecules of Runx2
Source: Commun Biol. 2021 Oct 19;4:1199. doi: 10.1038/s42003-021-02717-7 (PMC8526618; doi:10.1038/s42003-021-02717-7)
Supplement: Supplementary file 1 — Transparent Peer Review File [file 42003_2021_2717_MOESM1_ESM.pdf]

nature portfolio

# Peer Review File

## Smoc1 and Smoc2 regulate bone formation as downstream molecules of Runx2

**Open Access** This file is licensed under a Creative Commons Attribution 4.0 International License, which permits use, sharing, adaptation, distribution and reproduction in any medium or format, as long as you give appropriate credit to the original author(s) and the source, provide a link to the Creative Commons license, and indicate if changes were made. In the cases where the authors are anonymous, such as is the case for the reports of anonymous peer reviewers, author attribution should be to 'Anonymous Referee' followed by a clear attribution to the source work. The images or other third party material in this file are included in the article's Creative Commons license, unless indicated otherwise in a credit line to the material. If material is not included in the article's Creative Commons license and your intended use is not permitted by statutory regulation or exceeds the permitted use, you will need to obtain permission directly from the copyright holder. To view a copy of this license, visit http://creativecommons.org/licenses/by/4.0/.

Reviewers' comments:

Reviewer #1 (Remarks to the Author):

Smoc1 & Smoc2

This paper submitted by Takahata et. al. entitled “Smoc1 and Smoc2 regulate bone formation as novel downstream molecules of Runx2” and tried to illuminate function of Smoc1 and Smoc2 that is relatively unknown in skeletal tissue.

Major comments:

1. The skeletal phenotype of Smoc1 single KO mouse should be described before explaining double KO result.

- If it had previously reported by others, authors can summarize it briefly. Then authors can raise specific questions for this paper.
- Or authors probably can present the result.
- It is important because Smoc2-/- single KO study indicated that it is not critical in osteogenesis. In previous report, Smoc1 null mouse also show a small body size, eye and limb phenotypes, and they died in 3 weeks. These results indicated that Smoc1 single KO may have a significant bone phenotype that could not be compensated by Smoc2. Authors can provide a good reasoning in the text for this point.

2. Smoc1 and Smoc2 double KO mouse showed mild decrease in long bone size but the severest phenotype is in under developed craniofacial tissues. The main problem seems not in the mineralization (that is found in Runx2 KO mice) but in craniofacial morphogenesis. To understand the problem better, it would be good to check later stage of mouse development (E18.5 or just after birth) in Fig. 4. It would be much better to include Smoc1+/-;Smoc2-/- group because double KO craniofacial morphogenesis is too severe.

3. Fig.1a. & 1b demonstrated that BMP2 over-expression showed much stronger induction of Smoc1 and Smoc2 than that by Runx2 over-expression, which indicate BMP2 has alternative ways to stimulate Smoc1 and Smoc2 expression that is not mediated by Runx2. In addition supplementary Fig. 3a & 3b also indicated Runx2 and BMP2 are not in the linear pathway to induce Smoc1 or Smoc2. Stimulation of Smoc expression by Runx2 seems only active in the presence of BMP2 treatment. Some mediator of BMP2 signaling such as Smad1&5 or Hox protein interaction with Runx2 could be suspected. There is a report that HoxC11 overexpression shows loss of fibula in mice. Could it be something to do with Smoc1 KO or Smoc1 & Smoc2 double KO mice phenotype? Anyway, it would be necessary to check whether Smoc expression is regulated by Runx2-independent BMP2 downstream signals.

Minor comments: There are many typographical or editing errors.

1. There is no title or legend explaining Table 1.
2. Information or reference explaining DN-Runx2 would be required.
3. Label of X-axis in Fig. 1g. is missed.
4. Fig. 3C. Please change Smoc1 and Smoc2 in the X-axis to shSmoc1 and shSmoc2, respectively.
5. What is cause of death in Smoc1 single KO or Smoc1 & Smoc2 double KO mouse? Please provide suggestions from previous paper or author’s speculation.
6. Is it correct the notion in line 57“unknown Runx2 regulatory molecules”(unknown Runx2-target molecules??)

Reviewer #2 (Remarks to the Author):

This is an important report revealing the new molecular network regulating bone formation via Smoc1/Smoc2 and Runx2.

As Runx2 is a transcription factor that plays a central role in bone formation, it is of interest to determine whether these new downstream genes of Runx2 are direct or indirect targets. The author's team may clarify the molecular network in details or at least potential molecular cascades should be

discussed.
The phenotype that Smoc1 and Smoc2 double KO mice completely lacked skull formation is convincing, strongly supporting the authors' hypothesis. However, as the authors noted, the function of Smoc1 and Smoc2 remains unclear. An additional detailed discussion about the potential molecular mechanisms of Smoc1 and Smoc2 in bone formation should help the leaders to understand the importance of this finding.

Reviewer #3 (Remarks to the Author):

The work attempts to provide improved information on the functional role of SMOC1/2 proteins in skeletal development, using a combination of quantitative gene expression studies in limb-bud cells, in vitro assays to show effects on osteoblast differentiation, and some targeted mouse genetics to link these with bone development in vivo. It is a novel study, in as far as the authors reveal a genetic pathway that includes Bmp2 and Runx2 inducing expression of the paralogous Smoc genes: Smoc1 and Smoc2. They also give a phenotypic description of Smoc1;Smoc2 double knockout mice, which is also novel.

Specific comments:

1. No statistics are given for the microarray analysis (e.g. number of replicates, p-values, FDR etc.) It was not explained how this data was analysed. The data in Table 1 only shows eight genes and these are all very similar in their gene family/type, which is a somewhat surprising result. Are these the top genes or the only upregulated genes? It is essential to include a comprehensive list of the values for all genes included on the array somewhere in the manuscript. The GEO data was not accessible to this reviewer as the data is currently private and is scheduled to be released on Feb 20, 2020. No token was provided.
2. In the microarray analysis a FC of +1.46 and +1.26 for Smoc1 and Smoc2, respectively, seems very low compared to the data presented in Figure 1, where equivalent experiment shows FC of +3-8 for Smoc1 and +2-2.5 for Smoc2 with Bmp2 treatment. The relative levels of Smoc1 and Smoc2 in the qRT-PCR seem to be opposite to the Microarray (Smoc1>Smoc2 in qRT-PCR, whereas Smoc2>Smoc1 in microarray). Can the authors explain these differences?
3. No detail is given to confirm the targeting of the Smoc genes in the mutant mice presented. There were no analyses of the genetic loci, the mRNA, or the protein, all of which are essential to confirm correct targeting and generation of null alleles.
4. The Smoc2 knockout mouse presented in FigS5 is smaller than the WT and has an apparent craniofacial phenotype. This should be discussed in the manuscript?
5. Remarkably (given point 4, above), Smoc1+/+;Smoc2-/- are used as controls in Figure 4 for comparison to Smoc1-/-;Smoc2-/- samples (see also, point 15 below). Please justify this choice of control.
6. In the Smoc1+/+;Smoc2-/- heads shown in figure 4C, is it possible these also have a subtle craniofacial phenotype that was not identified in this analysis? Recent studies have shown that a genetic insertion at the Smoc2 gene locus is associated with craniofacial phenotypes in brachycephalic dog breeds (Marchant et al. Current Biology. 2017). Analysis of defects or changes in craniofacial structures should be performed, e.g. by micro-CT and morphometric analysis.
7. Do Runx2 KO embryos show loss or changes to Smoc1 and Smoc2 expression during embryonic development? This should be relatively simple to perform using in situ hybridisation probes that the authors have already show work in Figure 2. If the link between Runx2 to Smoc gene expression is direct, then there should be a noticeable change of Smoc gene expression in specific tissues.
8. Is there any evidence that Runx2 binds to the Smoc genomic loci, either at the promoter regions or cis-regulatory regions, and that it directly regulates their expression? In silico analyses could be used to identify any Runx2 motifs in these regions, and Chromatin immunoprecipitation analyses could confirm this experimentally.
9. What is the direct link of Smoc gene function to bone formation? Do they effect mesenchymal stem

cell differentiation into osteoblasts or chondrocytes? It would be useful to address this robustly in either embryos, or more thoroughly in the cell culture systems used.
10. Can the authors add DN-Runx2 and BMP2 in combination to the Limb Bud cells to confirm the direct relationship between these and Smoc gene expression. Do they still see increases in Smoc expression in this context?
11. The In Situ hybridisation images in figure 2 are of low resolution and difficult to interpret. In addition, they are not novel as several studies have previously shown Smoc1 developmental expression at E11.5 (Rainger et al. 2011, Abouzid et al, 2011), and also Smoc2. To make better use of this data, the specific expression of both paralogues should be clearly annotated in specific tissue regions, and compared to show where they overlap or are individually specific. These in situs may also be somewhat over-exposed, as the limb bud expression does not clearly show the anterior-posterior specific expression of Smoc1 and its absence in the regions adjacent to the AER. Could the limbs be removed from the embryos to more clearly show the specific expression patterns and reduce some shadowing from the rest of the embryo body?
12. I was unsure of the relevance of Col2A1 in situ hybridisation in Figure 2. How does this relate to Smoc/Runx2 or Bmp2? A statement is required to provide this link more clearly.
13. There are no scale bars on the in situ images. The E11.5 embryos look older and I think these may not be accurately staged.
14. Quantitative analyses of the Alkaline phosphatase assays are required (Fig 3e-f). These images are currently insufficient to draw any meaningful conclusions from alone and the authors interpretation is therefore subjective.
15. There is inadequate information and phenotypic analysis of the mouse crosses. No metrics are given for the incidence of viable embryos for each genotype and penetrance rates of each phenotype are not given. It is important to know if the Smoc1+/-;Smoc2-/- displayed any phenotype, for example or how viable these were. Indeed, it was also stated that Smoc1-KO animals did not survive to weaning, but we are not given numbers for this statement. Robust analyses of all genotypes should be provided. How many mice were analysed?
16. For the bone phenotypes shown in Fig4, what was the range of measurements for these samples? These should be analysed quantitatively and compared to WT or heterozygotes and compound heterozygotes, not just to Smoc2-KO samples.
17. Labelling of anatomy is lacking in Fig4.
18. The phenotypes revealed in Fig4 are likely to have arisen during patterning events in early development, and preceeding ossification. What does the data actually tell us about the role of Smoc proteins in bone development, and how is this linked to Runx2 and Bmp2?
19. The data in Figure 5 is not convicing. There is no quantitation of signal, and the specimens shown are of poor quality and/or of low resolution. What does it mean if there is less signal in a tissue that is smaller than its control? These are largely phenomena and do not give any insight into mechanisms of bone formation mediated by Smoc proteins. This study would be more useful if performed at earlier stages of embryonic development.
20. Was Runx2 mRNA affected in Bmp2-treated mouse limb bud cells? What is the Runx2 expression when profiled against Smoc1/2 mRNA expression? What does the expression of these genes look like in whole mount in situ for the three knock-out lines?
21. Can the authors explain why the levels of Smoc1 mRNA is lover in Runx2 treated cells compared to Bmp2-treated cells? This seems surprising if the central tenet of this study is that Runx2 mediates Smoc gene expression.
22. Adding Bmp2 to cells also treated with DN-Runx2 would augment the data showing that Runx2 directly mediates Bmp2 influence on Smoc gene expression, adding to the data from the Runx2 KO mice (Fig1 g-h).
23. Are Osterix and Osteocalcin also upregulated in this system? Can the authors therefore directly link Smoc function to bone formation?
24. Figure 1f is not labelled. Figure 1g requires an axis legend.
25. Sample sizes are not provided on an experiment-by experiment basis, which is important to interpret the data shown.

**Reply to Reviewer 1**

We greatly thank the reviewer for reviewing our manuscript and providing us with constructive and important comments on our study.

*1. The skeletal phenotype of Smoc1 single KO mouse should be described before explaining double KO result.*

*- If it had previously reported by others, authors can summarize it briefly. Then authors can raise specific questions for this paper.*

*- Or authors probably can present the result.*

*- It is important because Smoc2-/- single KO study indicated that it is not critical in osteogenesis. In previous report, Smoc1 null mouse also show a small body size, eye and limb phenotypes, and they died in 3 weeks. These results indicated that Smoc1 single KO may have a significant bone phenotype that could not be compensated by Smoc2. Authors can provide a good reasoning in the text for this point.*

Reply: Although we described the previous reports regarding *Smoc1* mutant mice briefly in the original manuscript, we agree with the reviewer's comments that we need to provide greater details regarding these studies. Okada *et al.*, 2011 had previously reported that they created three lines of Smoc1 mutant mice. Lines 1 and 3 were backcrossed with C57BL6/J for at least four generations. Line 2 was maintained with mixed background C57BL6/J and ICR. The authors mainly analyzed line 1 in their study. Heterozygous mutant mice were healthy and fertile. Homozygous mice were viable at P0, but they did not survive the first 3 weeks. Their growth was retarded compared with WT and heterozygous littermates at P0 and P14. A clear phenotype of developmental defects in the eyes and optic nerves was observed at E14.5. Tibia and fibula of homozygous mice appeared mispositioned in both lines 1 and 2. Limb malformation were observed in line 2 but not in line 1. By contrast, another group, Rainger *et al*. also created *Smoc1* mutant mice. Their *Smoc1* mutant mice displayed similar phenotypes as our *Smoc1* mutant mice. Our *Smoc1* mutant mice shown in the present study were backcrossed with C57BL6/J for at least 10 generations, and die at birth, not surviving for more than 1 day. We also observed defects of eye development, mispositioned femur/fibula, and hindlimb malformation. We assume that some different phenotypes in *Smoc1* mutant mice occur between the previous literature and our study as a result of the background of the mice. Because *Smoc2* expression determined in the limb bud was very weak by whole-mount in situ hybridization (Fig. 2e), we

speculate that our *Smoc2* mutant mice did not display a clear phenotype in long bones. Because both, *Smoc1* and *Smoc2* were highly expressed in calvaria, a clear skeletal phenotype in the skull was not seen in *Smoc1* single KO mice, presumably because of Smoc2 compensation. In accordance with the reviewer's suggestion, we have described these important points in the revised manuscript by providing additional and new experimental data (Fig. 2, and Supplementary Fig. 5).

*2. Smoc1 and Smoc2 double KO mouse showed mild decrease in long bone size but the severest phenotype is in under developed craniofacial tissues. The main problem seems not in the mineralization (that is found in Runx2 KO mice) but in craniofacial morphogenesis. To understand the problem better, it would be good to check later stage of mouse development (E18.5 or just after birth) in Fig. 4. It would be much better to include Smoc1+/-;Smoc2-/- group because double KO craniofacial morphogenesis is too severe.*

Reply: We thank the reviewer for these important comments. To address these points, we mated *Smoc1*(+/−);*Smoc2*(+/−) mice with each other, and analyzed the skeletal phenotypes of all the genotypes in *Smoc1* and *Smoc2* mutant mice at the E18.5 stage (Supplementary Fig. S11). Severe defects of craniofacial morphogenesis were observed in both, *Smoc1* and *Smoc2* double KO (*Smoc1*−/−;*Smoc2*−/−) mice and *Smoc1*(−/−);*Smoc2*(+/−) mice. By contrast, *Smoc1*(+/−);*Smoc2*(−/−) mice displayed a normal skeletal phenotype with intact calvaria formation and were fertile similar to WT mice. These new data are included in the revised manuscript. We believe that the different phenotypes in the calvarias are due to the *Smoc1* and *Smoc2* expression levels and different expression patterns within tissues of *Smoc1* and *Smoc2*. We appreciate the reviewer's excellent comments, such that we could more deeply investigate the roles of Smoc1 and Smoc2.

*3. Fig.1a. & 1b demonstrated that BMP2 over-expression showed much stronger induction of Smoc1 and Smoc2 than that by Runx2 over-expression, which indicate BMP2 has alternative ways to stimulate Smoc1 and Smoc2 expression that is not mediated by Runx2. In addition supplementary Fig. 3a & 3b also indicated Runx2 and BMP2 are not in the linear pathway to induce Smoc1 or Smoc2. Stimulation of Smoc expression by Runx2 seems only active in the presence of BMP2 treatment.*

Reply: We thank the reviewer for the valuable suggestion. In response to the reviewer's comment, we carefully and extensively repeated the experiments, and confirmed that *Smoc1* and *Smoc2* expressions were sufficiently induced by infection with Runx2 adenovirus after 4–6 days of culture (Fig. 1,

Supplementary Table 2). Moreover, we additionally performed ChIP assays, and found the direct binding of Runx2 to the *Smoc1* and *Smoc2* gene promoters (Supplementary Fig. 4). Therefore, we believe that Runx2 directly regulates *Smoc1* and *Smoc2* expressions. However, we observed that DN-Runx2 did not completely inhibit Bmp2-induced *Smoc1* and *Smoc2* expressions in both, WT and *Runx2*-deficient cells (Fig. 1i, j). Additionally, following a short (24 h) Runx2 and/or Bmp2 adenovirus infection, we found synergistic effects of Runx2 and Bmp2 on *Smoc1* and *Smoc2* expressions (Supplementary Fig. 3). Therefore, although our new data support our hypothesis that Runx2 directly regulates *Smoc1* and *Smoc2* expressions, a Runx2-independent pathways might be involved in BMP2-induced *Smoc1* and *Smoc2* expressions as the reviewer indicated. We believe that our study clearly demonstrates our major purpose of this study to identify the Runx2 downstream targets necessary for skeletal development and the reviewer's incisive comments greatly improved our study. We thank the reviewer for giving us the opportunity to pursue a deeper investigation in our study.

*-Some mediator of BMP2 signaling such as Smad1&5 or Hox protein interaction with Runx2 could be suspected. There is a report that HoxC11 overexpression shows loss of fibula in mice. Could it be something to do with Smoc1 KO or Smoc1 & Smoc2 double KO mice phenotype? Anyway, it would be necessary to check whether Smoc expression is regulated by Runx2-independent BMP2 downstream signals.*

Reply: According to the reviewer's excellent suggestion, we performed additional experiments and examined the involvement of Hoxc11 in the regulation of *Smoc1* and *Smoc2* expressions. We demonstrated that Bmp2 induced *Hoxc11* mRNA expression (Supplementary Fig 12a). We also found an association of Hoxc11 with Runx2 by performing co-immunoprecipitation experiments (Supplementary Fig. 12b). However, Hoxc11 did not affect *Smoc1* or *Smoc2* expressions (Supplementary Fig. 12c and d). Therefore, Hoxc11 is not involved in Runx2-independent *Smoc1* or *Smoc2* induction, and other molecules would be involved in BMP2-induced *Smoc1* and *Smoc2* expressions, although the identification of these is not our aim in the present study. We greatly appreciate the important suggestion that improved our study.

*Minor comments: There are many typographical or editing errors.*

We are very sorry for our errors and thank the reviewer for the careful reading of the original manuscript.

*1. There is no title or legend explaining Table 1.*

Reply: We replaced Table 1 of the microarray screening data with RNA-sequence data that were newly obtained to confirm our original data (Supplementary Fig. 1, Supplementary Tables 1 and 2), and added the legend in the revised manuscript.

*2. Information or reference explaining DN-Runx2 would be required.*

Reply: The dominant-negative Runx2 (DN-Runx2) used in this study consisted of the amino acids 2–247 of Runx2 tagged with Flag epitope. This construct lacks the transcription activation domain at the C-terminal region. We added this information in the revised manuscript.

*3. Label of X-axis in Fig. 1g. is missed.*

Reply: We added a label to the X-axis in the revised manuscript.

*4. Fig. 3C. Please change Smoc1 and Smoc2 in the X-axis to shSmoc1 and shSmoc2, respectively.*

Reply: We corrected the labels of the X-axis to shSmoc1 and shSmoc2 in Fig. 3c.

*5. What is cause of death in Smoc1 single KO or Smoc1 & Smoc2 double KO mouse? Please provide suggestions from previous paper or author's speculation.*

Reply: We currently do not know the reason why *Smoc1* KO mice are lethal. We believe that *Smoc1* and *Smoc2* double KO mice are unable to survive because of the severe craniofacial phenotype, which exposes the brain parenchyma.

*6. Is it correct the notion in line 57"unknown Runx2 regulatory molecules"(unknown Runx2-target molecules??)*

Reply: We are sorry for giving this misleading description. We made the correction as pointed out by the reviewer to avoid any misunderstanding by the readers.

**Reply to Reviewer 2**

*This is an important report revealing the new molecular network regulating bone formation via Smoc1/Smoc2 and Runx2.*

*As Runx2 is a transcription factor that plays a central role in bone formation, it is of interest to determine whether these new downstream genes of Runx2 are direct or indirect targets. The author's team may clarify the molecular network in details or at least potential molecular cascades should be discussed.*

*The phenotype that Smoc1 and Smoc2 double KO mice completely lacked skull formation is convincing, strongly supporting the authors' hypothesis. However, as the authors noted, the function of Smoc1 and Smoc2 remains unclear. An additional detailed discussion about the potential molecular mechanisms of Smoc1 and Smoc2 in bone formation should help the leaders to understand the importance of this finding.*

Reply: We greatly thank the reviewer for critically reviewing our manuscript and providing us with constructive comments. We are very grateful that the reviewer evaluated our study as important and interesting. As the reviewer suggests, it is important to know whether Smoc1 and Smoc2 are direct or indirect targets of Runx2. We conducted additional experimental RNA sequence analyses and repeated the RT-qPCR analyses, and consequently found that Runx2 alone sufficiently induced Smoc1 and Smoc2 expressions (Fig. 1a and 1b, Supplementary Table 2). Moreover, we found that direct binding of Runx2 to the Smoc1 and Smoc2 gene promoters occurred by performing ChIP assays (Supplementary Fig. 4). In addition, we performed whole-mount *in situ* hybridization using *Runx2* KO mice and found that *Smoc1* tended to decrease in systemic expression, while *Smoc2* expression was suppressed in the skull of the mice (Fig. 2). Therefore, we believe that Runx2 directly regulates *Smoc1* and *Smoc2* expressions. However, because the effect of DN-Runx2 did not completely suppress Bmp2-dependent *Smoc1* and *Smoc2* expressions (Fig.1i and j), it is possible that Runx2-independent downstream signals are also involved in the *Smoc1* and *Smoc2* induction. We described this possibility in the revised manuscript. Although it is not our major scope to understand the precise roles of Smoc1 and Smoc2 in skeletal development, we describe their potential roles in skeletal development in the revised manuscript. We appreciate the valuable comments.

**Reply to Reviewer 3**

*The work attempts to provide improved information on the functional role of SMOC1/2 proteins in skeletal development, using a combination of quantitative gene expression studies in limb-bud cells, in vitro assays to show effects on osteoblast differentiation, and some targeted mouse genetics to link these with bone development in vivo. It is a novel study, in as far as the authors reveal a genetic pathway that includes Bmp2 and Runx2 inducing expression of the paralogous Smoc genes: Smoc1 and Smoc2. They also give a phenotypic description of Smoc1;Smoc2 double knockout mice, which is also novel.*

We greatly thank the reviewer for critically reviewing our manuscript and providing us with constructive and important comments. We are also very pleased that the reviewer evaluated our findings as novel and providing improved information on the functional role of Smoc1/2 on skeletal development.

Specific comments:

*1. No statistics are given for the microarray analysis (e.g. number of replicates, p-values, FDR etc.) It was not explained how this data was analysed. The data in Table 1 only shows eight genes and these are all very similar in their gene family/type, which is a somewhat surprising result. Are these the top genes or the only upregulated genes? It is essential to include a comprehensive list of the values for all genes included on the array somewhere in the manuscript. The GEO data was not accessible to this reviewer as the data is currently private and is scheduled to be released on Feb 20, 2020. No token was provided.*

Reply: We thank the reviewer for providing us with these important comments. We deeply apologize that the reviewer was unable to access the GEO data. In this study, we performed microarray analysis solely for the purpose of initial screening for genes that are upregulated by Bmp2, which regulates Runx2 expression and function. Therefore, the initial microarray was performed as a single screening experiment in the original manuscript. To confirm the original data and analyze it, we additionally performed RNA-sequence analyses with duplicate samples. Therefore, we replaced the original microarray data with the new RNA-sequence analyses data (Supplementary Fig. 1, Supplementary Tables 1 and 2). In response to the reviewer's suggestion that a comprehensive list should be provided, we added this list in the revised manuscript (Supplementary Tables 1 and 2). We deposited the data in

the NCBI database and will forward the token to the editorial office for the reviewer to access the original data.

*2. In the microarray analysis a FC of +1.46 and +1.26 for Smoc1 and Smoc2, respectively, seems very low compared to the data presented in Figure 1, where equivalent experiment shows FC of +3-8 for Smoc1 and +2-2.5 for Smoc2 with Bmp2 treatment. The relative levels of Smoc1 and Smoc2 in the qRT-PCR seem to be opposite to the Microarray (Smoc1>Smoc2 in qRT-PCR, whereas Smoc2>Smoc1 in microarray). Can the authors explain these differences?*

Reply: We thank the reviewer for these important comments. As described above, we additionally performed RNA-sequence analyses and replaced the data. Although we performed microarray analysis for first screening purposes, we seriously considered that microarray analysis might be insufficient to guarantee accurate quantification because of the varying chip quality on RNA hybridization. Our new RNA sequence analyses indicate a *Smoc1* 3.81-fold increase by Runx2 vs Venus, a *Smoc1* 3.01-fold increase by Bmp2 vs Venus, a *Smoc2* 2.07-fold increase by Runx2 vs Venus, and a *Smoc2* 5.16-fold increase by Bmp2 vs Venus (Supplementary Tables 1 and 2). In addition, we statistically determined the induction of *Smoc1* and *Smoc2* expressions by Runx2 and Bmp2 by RT-qPCR. ($n$ = 4–6) (Fig. 1a, b). To confirm the data, we extensively repeated RT-qPCR analyses as shown in the new Figure 1 for accurate quantitative evaluation. Additionally, we assume that the differences of the data between microarray analysis and RT-qPCR in the original manuscript were due to methodological differences between the two assays. Overall, we could confirm our results, and appreciate the reviewer's excellent comments.

*3. No detail is given to confirm the targeting of the Smoc genes in the mutant mice presented. There were no analyses of the genetic loci, the mRNA, or the protein, all of which are essential to confirm correct targeting and generation of null alleles.*

Reply: We apologize for not providing the data required for the confirmation of the mutation of the mice. In this study, we confirmed the mutation of the *Smoc1* and *Smoc2* genes in the heterozygous mutant mice by performing Southern blotting and PCR analyses with genomic DNA. We also confirmed the deletion of the *Smoc1* and *Smoc2* genes in the *Smoc1*- and *Smoc2*-deficient mice, respectively, by performing PCR analyses with genomic DNA. We incorporated the data in the revised manuscript (Supplementary Figs. 6 and 7). To address the reviewer's question, we examined *Smoc1*

and *Smoc2* expressions by performing RT-qPCR analyses using *Smoc1* and *Smoc2* KO mice, and confirmed the deletion of their expressions in the mice. We also incorporated the data in the revised manuscript (Supplementary Figs. 6c and 7c).

*4. The Smoc2 knockout mouse presented in FigS5 is smaller than the WT and has an apparent craniofacial phenotype. This should be discussed in the manuscript?*

Reply: We apologize for this misleading result. According to previous literature (Marchant et al. Current Biology, 2017), retrotransposon-mediated missplicing of the *SMOC2* gene can cause brachycephaly in dogs. Therefore, we carefully quantified the length from the nasal bone to the parietal bone of both WT and *Smoc2* KO mice at the E18.5 stage. We found that this length in the KO mice was significantly shorter than in WT mice. In addition, we analyzed micro-CT images of 8-week-old *Smoc2* KO mice. It was also revealed that *Smoc2* KO mice tend to have brachycephaly compared with WT mice. Although *Smoc2* KO mice grow normally and maintain fertility, it was found that there are slight changes in the craniofacial morphogenesis of Smoc2 KO mice. We added the micro-CT data (Supplementary Fig. 9g) and the data of the quantified length (Supplementary Fig. 9h, i) in the revised manuscript. We greatly appreciate the reviewer's suggestions that have strengthened and improved our study.

*5. Remarkably (given point 4, above), Smoc1+/+;Smoc2-/- are used as controls in Figure 4 for comparison to Smoc1-/-;Smoc2-/- samples (see also, point 15 below). Please justify this choice of control.*

Reply: A mild craniofacial phenotype was observed in *Smoc2* KO mice, but no change was seen in the long bone. Therefore, we used *Smoc2* single KO mice as a control to examine the role of *Smoc1* and *Smoc2* in the *Smoc2*-deficient background. In addition, we have now considered the reviewer's very important comment. Indeed, we observed the mild phenotype in the *Smoc2* KO mice as described above (point #4). We then mated *Smoc1*(+/−);*Smoc2*(+/−) mice with each other, and analyzed every genotype pattern in *Smoc1* and *Smoc2* mutant mice at the E18.5 stage (Supplementary Fig. 11, and Supplementary Table 3). Severe defects of craniofacial morphogenesis were observed in *Smoc1*(−/−-);*Smoc2*(+/−) mice in addition to *Smoc1* and *Smoc2* double KO (*Smoc1*−/−; *Smoc2*−/−) mice, but not in *Smoc1*(+/−);*Smoc2*(−/−) mice. By contrast, *Smoc1*(+/−);*Smoc2*(−/−) mice displayed a very similar skeletal phenotype with *Smoc1*(+/+);*Smoc2*(−/−) mice. Regarding the reviewer's point

15 below, we added whole skeletal appearance of all genotypes in Supplementary Fig. 11 in the revised manuscript. We also incorporated the data of the occurrence of phenotypes in all genotypes (Supplementary Table 3). We thank the reviewer so much for encouraging us to determine the phenotype of the double mutant mice more precisely and adequately.

*6. In the Smoc1+/+;Smoc2-/- heads shown in figure 4C, is it possible these also have a subtle craniofacial phenotype that was not identified in this analysis? Recent studies have shown that a genetic insertion at the Smoc2 gene locus is associated with craniofacial phenotypes in brachycephalic dog breeds (Marchant et al. Current Biology. 2017). Analysis of defects or changes in craniofacial structures should be performed, e.g. by micro-CT and morphometric analysis.*

Reply: We thank the reviewer for the constructive comment. In response to the reviewer's comments 4 above and 6, we performed micro-CT analysis of 8-week-old *Smoc2* KO and WT mice (Supplementary Fig. 9g), and quantified the length of the nasal bone to the parietal bone at the E18.5 stage, and the craniofacial phenotype of brachycephaly was observed in *Smoc2* KO mice (Supplementary Fig. 9h and i). We incorporated and described the data in the revised manuscript as mentioned above.

*7. Do Runx2 KO embryos show loss or changes to Smoc1 and Smoc2 expression during embryonic development? This should be relatively simple to perform using in situ hybridisation probes that the authors have already show work in Figure 2. If the link between Runx2 to Smoc gene expression is direct, then there should be a noticeable change of Smoc gene expression in specific tissues.*

Reply: According to the reviewer's important and constructive comments, we performed whole-mount *in situ* hybridization using *Runx2* KO embryos as shown in Figure 2. *Smoc1* expression was observed in the skull, clavicle, and long bone at the E12.5 stage, whereas the expression in the limb bud was very weak at least at this stage (Fig. 2, Supplementary Fig. 5). We found that calvarial *Smoc1* expression in *Runx2* KO mice was moderately decreased compared with WT mice (Fig. 2a). Strong *Smoc2* expression was observed in the skull of WT mice (Fig. 2d), whereas very weak signals were detected in other tissues (Fig. 2e, f). Calvarial *Smoc2* expression in *Runx2* KO mice was clearly decreased compared with WT mice (Fig. 2d). These results support the data that *Smoc1* and *Smoc2* double KO mice display a severe phenotype of the craniofacial region and suggest the importance of

the association between Runx2 and Smoc1/2. We replaced the data in Fig. 2 with the newly obtained data, and incorporated additional data in Supplementary Fig. 5 in the revised manuscript.

*8. Is there any evidence that Runx2 binds to the Smoc genomic loci, either at the promoter regions or cis-regulatory regions, and that it directly regulates their expression? In silico analyses could be used to identify any Runx2 motifs in these regions, and Chromatin immunoprecipitation analyses could confirm this experimentally.*

Reply: The reviewer raised a very important and critical question. Runx2 consensus binding motifs are known as (Pu)PuCCACA and TGTGGPy(Py). We analyzed the published ChIP-seq data from GEO as described below. Based on this information, we performed ChIP assays, and found that Runx2 directly binds to *Smoc1* and *Smoc2* gene promoter regions and introns (Supplementary Fig. 4). With reference to these ChIP data, we confirmed that Runx2 protein binding sites are present on the *Smoc1* and *Smoc2* gene promoter regions. These data indicate that both Smoc1 and Smoc2 are direct downstream target molecules of Runx2. We incorporated the ChIP assay data in Supplemental Fig. 4 in the revised manuscript.

ID: SRX201981

Name: Runx2 (@ MC3T3-E1)

Title: GSM1027478: MC3T3 Runx2Veh; Mus musculus; ChIP-Seq

Cell group: Bone

Source_name: MC3T3-E1 cells

Passage number: 5 – 12

Antibody: RUNX2 (Santa Cruz, M-70, sc10758, lot# D1411)

Cell line: MC3T3-E1

Treatment: ethanol vehicle

*9. What is the direct link of Smoc gene function to bone formation? Do they effect mesenchymal stem cell differentiation into osteoblasts or chondrocytes? It would be useful to address this robustly in either embryos, or more thoroughly in the cell culture systems used.*

Reply: The reviewer asks an important question. In the original manuscript, we investigated the role of Smoc1 and Smoc2 in osteoblasts and demonstrated their importance in osteoblast differentiation

and maturation (Fig. 3). To address the role of Smoc1 and Smoc2 in osteoblast and chondrocyte differentiation of mesenchymal cells, we isolated limb buds cells as mesenchymal cells from *Smoc1* or *Smoc2* KO mice, and examined the effects of Smoc1 and Smoc2 on osteoblast and chondrocyte differentiation. *Smoc1*-deficient limb bud cells displayed decreased expression of the early chondrocyte marker genes *Col2a1* and *Sox9*, late chondrocyte and osteoblast marker gene *Mmp13*, and osteoblast marker genes *Osterix* and *Runx2*, but not *Col10a1* (Supplemental Fig. 13a). We also found a reduction in chondrogenic extracellular matrix components as determined by alcian blue staining (Supplemental Fig. 13b) and ALP activity (Supplemental Fig. 13c) in *Smoc1*-deficient cells. These *in vitro* analyses suggest the possibility that *Smoc1* has the ability to stimulate osteoblast and chondrocyte differentiation of mesenchymal cells *in vitro*. By contrast, no significant changes in osteoblast or chondrocyte differentiation marker genes expressions were observed in *Smoc2*-deficient limb bud cells (Supplementary Fig. 14). These data suggest that Smoc2 might not be involved in osteoblast or chondrocyte differentiation from mesenchymal cells. The inconsistency between these *in vitro* data and *in vivo* analyses (Figs. 4 and 5, Supplementary Figs. 10 and 11) might have resulted from distinct tissue expressions of *Smoc1* and *Smoc2 in vivo* and differences between *in vitro* and *in vivo* analyses. We incorporated the new data in Supplementary Figs. 13 and 14. We greatly thank the reviewer for alerting us to another aspect of Smoc1 and Smoc2.

*10. Can the authors add DN-Runx2 and BMP2 in combination to the Limb Bud cells to confirm the direct relationship between these and Smoc gene expression. Do they still see increases in Smoc expression in this context?*

Reply: According to the reviewer's suggestion, we extensively investigated the effect of DN-Runx2 on Bmp2-induced *Smoc1* and *Smoc2* expressions in limb bud cells. The Bmp2-induced *Smoc1* and *Smoc2* expression levels were significantly but incompletely suppressed by DN-Runx2 adenovirus in the WT cells (Fig. 1i, j). As expected, in *Runx2*-deficient limb bud cells, Bmp2 treatment did not increase *Smoc1* expression. By contrast, Bmp2 upregulated *Smoc2* expression in *Runx2*-deficient cells, and this Bmp2-induced upregulation was inhibited by DN-Runx2. Considering the results of the induction by Runx2 (Fig. 1a, b, Supplementary Table 2) and the ChIP assay (Supplementary Fig. 4), we believe that Smoc1 and Smoc2 are direct targets of Runx2. However, Runx2-independet pathways

might be involved in Bmp2-induced *Smoc1* and *Smoc2*, and the pathways appear to make different contributions to Smoc1 and Smoc2 expressions.

*11. The In Situ hybridisation images in figure 2 are of low resolution and difficult to interpret. In addition, they are not novel as several studies have previously shown Smoc1 developmental expression at E11.5 (Rainger et al. 2011, Abouzid et al, 2011), and also Smoc2. To make better use of this data, the specific expression of both paralogues should be clearly annotated in specific tissue regions, and compared to show where they overlap or are individually specific. These in situs may also be somewhat over-exposed, as the limb bud expression does not clearly show the anterior-posterior specific expression of Smoc1 and its absence in the regions adjacent to the AER. Could the limbs be removed from the embryos to more clearly show the specific expression patterns and reduce some shadowing from the rest of the embryo body?*

Reply: According to the reviewer's comments, we repeated whole-mount *in situ* hybridization experiments data. In particular, we also examined *Smoc1* and *Smoc2* expressions in *Runx2* KO mice by performing a whole-mount *in situ* hybridization experiment. To respond to the reviewer's request, we showed the limbs, calvariae, and vertebrae from the embryo bodies. We replaced the original data with the new data in Fig. 2 in the revised manuscript.

*12. I was unsure of the relevance of Col2A1 in situ hybridisation in Figure 2. How does this relate to Smoc/Runx2 or Bmp2? A statement is required to provide this link more clearly.*

Reply: We included the results of *Col2a1* expression as a positive control for chondrogenesis and a positive control of *in situ* hybridization experiments. We totally agree with the reviewer's opinion. To avoid any misunderstanding by the readers, we removed the data.

*13. There are no scale bars on the in situ images. The E11.5 embryos look older and I think these may not be accurately staged.*

Reply: Because the magnification of all the panels were the same in the original figure (Fig. 2), we did not add scale bars on all panels. We are very sorry for confusing the presentation. As described above (#12), we replaced the data and incorporated the scale bars in all the panels.

*14. Quantitative analyses of the Alkaline phosphatase assays are required (Fig 3e-f). These images are currently insufficient to draw any meaningful conclusions from alone and the authors interpretation is therefore subjective.*

Reply: According to the reviewer's comment, we additionally performed the experiments in which we quantitatively determined the alkaline phosphatase activity using p-nitrophosphate as a substrate. Alkaline phosphatase activity in osteoblasts infected with shSmoc1 or/and shSmoc2 was significantly decreased compared to that of the shGFP control. We incorporated Fig. 3f in the revised manuscript. We thank the reviewer for this important comment.

*15. There is inadequate information and phenotypic analysis of the mouse crosses. No metrics are given for the incidence of viable embryos for each genotype and penetrance rates of each phenotype are not given. It is important to know if the Smoc1+/-;Smoc2-/- displayed any phenotype, for example or how viable these were. Indeed, it was also stated that Smoc1-KO animals did not survive to weaning, but we are not given numbers for this statement. Robust analyses of all genotypes should be provided. How many mice were analysed?*

Reply: We agree with the reviewer's comments. To respond to the reviewer's comments, we mated *Smoc1*(+/−);*Smoc2*(+/−) mice with each other, and analyzed the numbers of all the genotypes at the E18.5 stage (Supplementary Fig. 11). The distribution was similar to what would be expected from Mendel's laws (Supplementary Table S3). Severe defects of craniofacial morphogenesis were observed in *Smoc1*(−/−); *Smoc2*(+/−) mice in addition to *Smoc1* and *Smoc2* double KO (*Smoc1*−/−; *Smoc2*−/−) mice. By contrast, *Smoc1*(+/−);*Smoc2*(−/−) mice displayed normal skeletal formation as mentioned in comment 15 above. Penetrance rates of each genotype were added in Supplementary Table 3 in the revised manuscript. We appreciate the excellent comments on the study and believe that the additional experiments strengthen and improve our study.

*16. For the bone phenotypes shown in Fig4, what was the range of measurements for these samples? These should be analysed quantitatively and compared to WT or heterozygotes and compound heterozygotes, not just to Smoc2-KO samples.*

Reply: Regarding comment 15 above, we examined all the genotypes of *Smoc1* and *Smoc2* mutant mice by crossing *Smoc1*(+/−);*Smoc2*(+/−) mice with each other. We added the numbers of all the

mutants we analyzed and incorporated the new data in Supplementary Table 3 in the revised manuscript.

*17. Labelling of anatomy is lacking in Fig4.*

Reply: We apologize for our careless errors, and labelled the anatomy in the revised figure. We thank the reviewer for such careful reading of the manuscript.

*18. The phenotypes revealed in Fig4 are likely to have arisen during patterning events in early development, and preceeding ossification. What does the data actually tell us about the role of Smoc proteins in bone development, and how is this linked to Runx2 and Bmp2?*

Reply: We believe that the data shown in Fig.4 indicate important roles of Smoc1 and Smoc2 in bone formation *in vivo*. To demonstrate the relationship of Runx2 and Bmp2 to the role of Smoc1 and Smoc2, we examined the effects of Smoc1 and Smoc2 on osteoblast differentiation and calcification, and found that these were strongly suppressed by shSmoc1 or/and shSmoc2 treatment, as shown in Fig. 3. *Smoc1* and *Smoc2* expressions were clearly observed in the skull at E12.5 (Fig. 2), which is the developmental stage of the skull. Moreover, the whole-mount *in situ* hybridization experiment results clearly showed reduced *Smoc1* and *Smoc2* expressions at the E12.5 stage in *Runx2* KO mice (Fig. 2). In addition, abnormal skull formation was observed at the earlier stages of E12.5 and E13.5 (Supplementary Fig. 10). While we agree with the reviewer's comment, we do believe that our *in vitro* and *in vivo* data collectively indicate the association of Runx2 and Bmp2 with the role of Smoc1 and Smoc2 shown in Fig. 4, and that the differences in the patterning effect would be partly involved in the phenotype.

*19. The data in Figure 5 is not convicing. There is no quantitation of signal, and the specimens shown are of poor quality and/or of low resolution. What does it mean if there is less signal in a tissue that is smaller than its control? These are largely phenomena and do not give any insight into mechanisms of bone formation mediated by Smoc proteins. This study would be more useful if performed at earlier stages of embryonic development.*

Reply: We apologize for any difficulties caused by the indistinct figures. The *in situ* hybridization diagram shown in Fig. 5 may have been compressed for the review process. Our original data are clear with good resolution and quality. As the reviewer indicated, the stained signal in a tissue is not

adequate for quantification. The purpose of *in situ* hybridization and von Kossa analyses are to determine which stages of endochondral bone formation are affected in the mutant mice. We are very confident of our *in situ* hybridization and von Kossa staining analyses and that the data clearly indicated that endochondral ossification of the mutant mice was impaired at the late stage. It is too early and not suitable to examine the phenotype of the mice at the earlier stages of E12.5, E13.5, or E14.5, when we cannot evaluate the late stage of endochondral ossification. We therefore performed histological analyses at E15.5.

To respond to the reviewer's request and ensure quantification, total RNA was extracted from the entire tibia, and the expressions of chondrocyte marker genes were determined by RT-qPCR. *Runx2* and *Osterix* expressions in the tibiae of *Smoc1*(−/−);*Smoc2*(−/−) mice were significantly decreased compared to those of *Smoc1*(+/+);*Smoc2*(−/−) mice (Fig. 5d). The quantitative analyses strongly support our histological analyses. We added the new RT-qPCR data in Fig. 5 in the revised manuscript. Although the *Col10a1* expression level was similar in WT and *Smoc1* and *Smoc2* DKO mice (Fig. 4d), the *Col10a1* expression pattern was quite different between them (Fig. 4c). It is also evident that endochondral bone formation was delayed at the late stage in the DKO mice.

*20. Was Runx2 mRNA affected in Bmp2-treated mouse limb bud cells? What is the Runx2 expression when profiled against Smoc1/2 mRNA expression? What does the expression of these genes look like in whole mount in situs for the three knock-out lines?*

<u>Reply:</u> To respond to the reviewer's comments, we examined *Runx2* expression in limb bud cells cultured with Bmp2. As shown in Supplementary Fig. 2, Bmp2 increased *Runx2* expression. Moreover, we examined the expression patterns of *Runx2*, *Smoc1*, and *Smoc2* in each of the three KO lines by performing whole-mount *in situ* hybridization. As shown in Supplementary Fig. 5, Runx2 expression in the long bone tended to decrease in *Smoc1* KO mice compared with WT mice. We did not observe any clear differences in *Smoc1* or *Smoc2* expressions in *Smoc2* KO or *Smoc1* KO mice, respectively. We assume that *Smoc1* and *Smoc2* deficiencies do not affect *Smoc2* or *Smoc1* expression, respectively (Supplementary Fig. 5). As described above, *Smoc1* and *Smoc2* expressions were reduced in the skull of *Runx2* KO mice (Fig. 2). Additionally, *Smoc1* or *Smoc2* deficiency did not alter *Runx2* expression. We incorporated these data in the revised manuscript (Supplementary Fig. 5). We greatly thank the reviewer for the excellent comments.

*21. Can the authors explain why the levels of Smoc1 mRNA is lover in Runx2 treated cells compared to Bmp2-treated cells? This seems surprising if the central tenet of this study is that Runx2 mediates Smoc gene expression.*

Reply: To respond to the reviewer's questions, we extensively repeated the RT-qPCR experiments, including more groups, and performed RNA-sequence analyses (Fig. 1 and Supplementary Tables 1 and 2). We confirmed that Runx2 reproducibly induced *Smoc1* and *Smoc2* expressions; however, the effects of Runx2 on these expressions were different from those of Bmp2. One possible explanation is that we cannot accurately compare the effects of the transcription factor (Runx2) with those of the ligand (Bmp2). Another reason would be Runx2-independent pathways, as described above. Together with the results of the ChIP assays (Supplementary Fig. 4) and whole-mount *in situ* hybridization experiments using *Runx2* KO mice (Fig. 2), we are confident of our major proposal that *Smoc1* and *Smoc2* are direct targets of Runx2. We thank the reviewer for these comments that strengthened and improved our study.

*22. Adding Bmp2 to cells also treated with DN-Runx2 would augment the data showing that Runx2 directly mediates Bmp2 influence on Smoc gene expression, adding to the data from the Runx2 KO mice (Fig1 g-h).*

Reply: In response to the reviewer's excellent suggestion, we additionally performed the experiments and examined the effect of Bmp2 on *Smoc1* and *Smoc2* expressions with or without DN-Runx2 adenovirus in limb bud cells isolated from WT or *Runx2* KO mice. As shown in Fig. 1i and 1j, DN-Runx2 suppressed the Bmp2-inducted *Smoc1* and *Smoc2* expressions. Because *Smoc2* expression in *Runx2* KO limb bud cells was increased by Bmp2 treatment, when responding to comment 21 above, Bmp2 also has possible roles in Smoc1 and Smoc2 expressions in Runx2-independnet manners.

*23. Are Osterix and Osteocalcin also upregulated in this system? Can the authors therefore directly link Smoc function to bone formation?*

Reply: We determined the expressions of *Osterix* and *osteocalcin*, both of which are well-known downstream molecules of Runx2, as positive controls to confirm the effects of Bmp2 treatment on the lumb bud cells. As expected, these genes were significantly upregulated in the Bmp2-treated group. Osterix is an important transcription factor for bone formation, and *Osterix* KO mice lack bone formation. By contrast, osteocalcin is a well-known marker for osteoblasts. Because *osteocalcin* KO

mice did not display impaired bone formation in a previous study (Ducy et al, *Nature*, 1996), osteocalcin does not appear essential for bone formation. Recently, Komori et al. (*Int J Mol Sci*, 2020) and Moriishi et al. (*PLoS Genet*, 2020) revealed a novel osteocalcin function, whereby it plays a role in the alignment of apatite crystallites. Because *Smoc2* is strongly expressed in the skull (Fig. 2) and calcification of osteoblasts is significantly suppressed by Smoc1 or/and Smoc2 knockdown in osteoblasts (Fig. 3), together with impaired bone formation of our mutant mice, Smoc1 and Smoc2 are considered to function in bone formation. However, we are unsure whether the function of Smoc1/2 is direct or indirect on bone formation, although it is not in the scope of the present study. We appreciate the reviewer's comment.

*24. Figure 1f is not labelled. Figure 1g requires an axis legend.*

Reply: We apologize for our careless errors. We replaced the original figure with new data and added the label to the axis in the revised manuscript.

*25. Sample sizes are not provided on an experiment-by experiment basis, which is important to interpret the data shown.*

Reply: We are very sorry for not providing the sample numbers. We now describe the sample size for each experiment in the figure legends.

Reviewers' comments:

Reviewer #3 (Remarks to the Author):

The authors have performed a tremendous amount of additional work, and the study is greatly improved. I have some remaining comments I feel would be important to address:
1) I welcome the RNAseq data, however the analysis is insufficiently described and not enough data has been presented. Please provide PCA analysis and more information on read quality counts. What were the criteria for inclusion/rejection of reads. Also, the supplemental legend to figure 1 refers to a LFC of >2, whereas the figure and main text use LFC>1. There are no metrics for adjusted p values, or what criteria were used to define down-regulated gene expression. This part of the study required significant supporting information to be included.
2) The discussion ends rather negatively (lines 297-305) and does not sufficiently convey the important finding of the work: the established genetic relationship between Bmp2-Runx2-Smoc1/2 in bone formation mechanisms. Could the authors provide a schematic to show this relationship, and include the downstream genes in this (e.g. Osterix, Col2a1, Sox9) and the Smoc-paralogue specificity observed.
3) The mouse genetics are a key part of this work and there should be more data given to show the targeting strategy. Currently, Supplemental figures 6&7 do not show the Smoc loci in sufficient detail. Specific exons need to be annotated and precise chromosomal positions should be indicated also. Both of these figures could be supported by some sequence data, or the Southern blot data that was mentioned as complete in the author's response (Comment #3).
4) The discussion may be improved by more in-depth analysis of the Smoc-Bmp genetic relationship and downstream mechanisms affected, perhaps in other developmental contexts. Also, as Smoc1 and Smoc2 have temporally different expression (Smoc1 is generally embryonic-early, Smoc2 embryonic-late), can the authors comment on possible other inducers of Smoc gene expression in other tissue contexts? For example, Bmp ligands are regionally and temporally expressed in the eye, and these are mostly Bmp4&7 and are dorsally located. In contrast, Smoc1 expression is ventral-specific. Similarly in the early limb bud Smoc1 is regionally restricted to the dorsal and ventral surface regions, while in the developing palate there are similar restrictions to Smoc expression. Can the authors speculate what the mechanisms of Smoc expression in relation to Bmp localisation are in these tissues?

To Reviewer 1's points:

The reviewer's comments has been sufficiently addressed. They now show the data requested for all of the genotypes and at the developmental stage requested. The data is well presented in Fig.S11.

However, the new data needs a little more explanation and detail. For example, I think the new Smoc1(-/-)Smoc2(+/-) craniofacial phenotype is novel and requires some quantitation, similar to the micro-CT in supplementary figure 11. This is important as the Smoc1 null mice apparently do not have this phenotype and it suggests that there is compensation between the Smocs, but this is insufficient for normal craniofacial development in a Smoc1-/-,Smoc2+/- genotype. They should also include a table indicating the penetrance of the phenotypes for each genotype, and at least show how many animals were observed with the phenotypes described. I could only find this for the calvaria phenotype but not the craniofacial defect.

Having gone over this again, it is my opinion that they should also label figure S9 panel G to indicate the measured points with respect to the CT scans.

Leading on from these comments, I don't know if this journal is a signatory to the ARRIVE Guidelines for animal use in research, but the data and study design do not appear to meet the essential 10 (https://arriveguidelines.org/arrive-guidelines). I'll let you decide on the importance of this.

**Reply to Reviewer 3**

We greatly thank the reviewer for reading our revised manuscript and providing valuable comments. We are pleased that the reviewer considered our revised manuscript to be greatly improved. Furthermore, we greatly appreciate the reviewer's effort to evaluate our changes in response to the comments of Reviewer 1.

*1) I welcome the RNAseq data, however the analysis is insufficiently described and not enough data has been presented. Please provide PCA analysis and more information on read quality counts. What were the criteria for inclusion/rejection of reads. Also, the supplemental legend to figure 1 refers to a LFC of >2, whereas the figure and main text use LFC>1. There are no metrics for adjusted p values, or what criteria were used to define down-regulated gene expression. This part of the study required significant supporting information to be included.*

Reply: We thank the reviewer for these important comments. Accordingly, we carefully checked the quality of our RNA-Seq data. We conducted PCA analysis of the data and confirmed the similarities within each group. We included the PCA analysis data in Supplementary Fig. 1a; our findings show that variance values clearly differ among Venus, Bmp2, and Runx2 groups. We also included the quality check data for each group following Fastqc analysis, as shown below. If the reviewer considers it necessary to incorporate these data in the manuscript, we are willing to include them as supplementary data.

Regarding the description of LFC, we apologize for confusion. In the revised manuscript, we ensured uniformity between the main text and the legend of Supplementary Figure 1 by using the phrase "log2 fold-change value > 1." Genes with log2 fold-change values > 1 were considered upregulated, while genes with log2 fold-change values < -1 were considered downregulated. We have included this information in the main text and the legend of Supplementary Figure 1. Importantly, we did not perform trimming of sequenced reads for mapping to the mouse reference genome; adjusted p-values were analyzed by iDEP9.1, in accordance with a published method (43).

# Venus Group:

## Basic Statistics

| Measure | Value |
|---|---|
| Filename | L.B_Venus_1_R1.fastq.gz |
| File type | Conventional base calls |
| Encoding | Sanger / Illumina 1.9 |
| Total Sequences | 20911325 |
| Sequences flagged as poor quality | 0 |
| Sequence length | 101 |
| %GC | 52 |

## Per base sequence quality

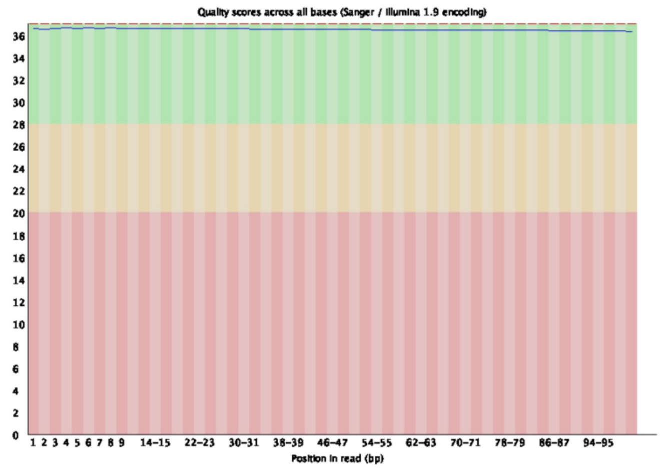


# BMP2 Group:

## Basic Statistics

| Measure | Value |
|---|---|
| Filename | L.B_Bmp2_1_R1.fastq.gz |
| File type | Conventional base calls |
| Encoding | Sanger / Illumina 1.9 |
| Total Sequences | 19633651 |
| Sequences flagged as poor quality | 0 |
| Sequence length | 101 |
| %GC | 51 |

## Per base sequence quality

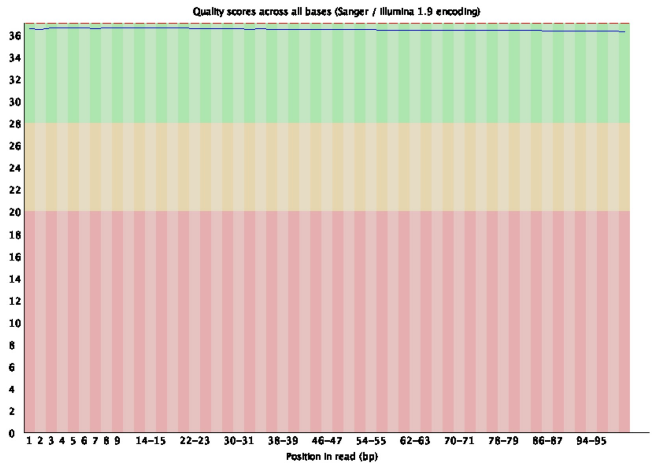

**Runx2 Group:**

**Basic Statistics**

| Measure | Value |
|---|---|
| Filename | L.B_Runx2_1_R1.fastq.gz |
| File type | Conventional base calls |
| Encoding | Sanger / Illumina 1.9 |
| Total Sequences | 22219248 |
| Sequences flagged as poor quality | 0 |
| Sequence length | 101 |
| %GC | 52 |

**Per base sequence quality**

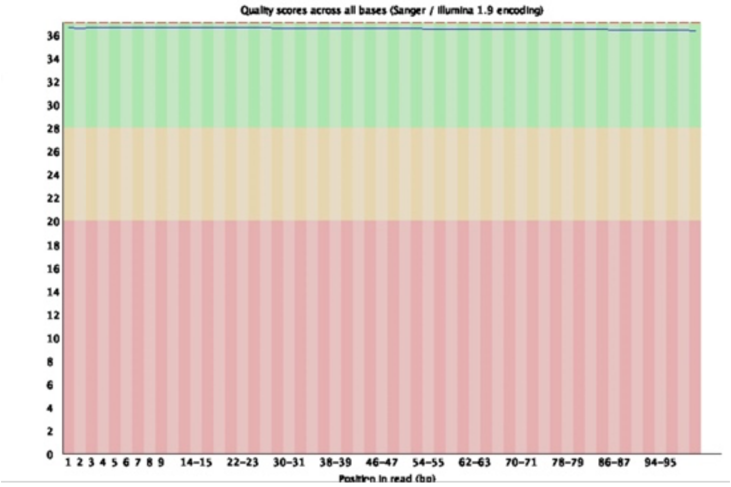


*2) The discussion ends rather negatively (lines 297-305) and does not sufficiently convey the important finding of the work: the established genetic relationship between Bmp2-Runx2-Smoc1/2 in bone formation mechanisms. Could the authors provide a schematic to show this relationship, and include the downstream genes in this (e.g. Osterix, Col2a1, Sox9) and the Smoc-paralogue specificity observed.*

Reply: We thank the reviewer for this meticulous assessment. Accordingly, we rewrote the last part of the Discussion in the revised manuscript and incorporated a new schematic figure to show the roles of the Bmp2-Runx2-Smoc1/2 axis in bone formation (Fig. 6). Because we do not have a clear concept of a Smoc-paralogue and we do not want to provide incorrect information, we did not discuss this aspect in the revised manuscript.

*3) The mouse genetics are a key part of this work and there should be more data given to show the targeting strategy. Currently, Supplemental figures 6&7 do not show the Smoc loci in sufficient detail. Specific exons need to be annotated and precise chromosomal positions should be indicated also. Both of these figures could be supported by some sequence data, or the Southern blot data that was mentioned as complete in the author's response (Comment #3).*

Reply: We thank the reviewer for the important comments. Although we provided details

regarding the precise chromosomal positions of Smoc1 and Smoc2 in the previous version of the Methods section, we added the chromosomal positions in the revised versions of Supplementary Figures 6 and 7. Moreover, we added Southern blotting data concerning Smoc1 flox mutant mice and Smoc2 mutant mice in Supplementary Figures 6b and 7b. We confirmed germline transmission of the mutant allele in the Smoc1 flox mutant and Smoc2 mutant mice upon establishment of both mutant mice. We included the sequences of the probes for Smoc1 and Smoc2 in the Supplementary information.

*4) The discussion may be improved by more in-depth analysis of the Smoc-Bmp genetic relationship and downstream mechanisms affected, perhaps in other developmental contexts. Also, as Smoc1 and Smoc2 have temporally different expression (Smoc1 is generally embryonic-early, Smoc2 embryonic-late), can the authors comment on possible other inducers of Smoc gene expression in other tissue contexts? For example, Bmp ligands are regionally and temporally expressed in the eye, and these are mostly Bmp4&7 and are dorsally located. In contrast, Smoc1 expression is ventral-specific. Similarly in the early limb bud Smoc1 is regionally restricted to the dorsal and ventral surface regions, while in the developing palate there are similar restrictions to Smoc expression. Can the authors speculate what the mechanisms of Smoc expression in relation to Bmp localisation are in these tissues?*
Reply: We thank the reviewer for this insightful comment. We agree that Smoc1 and Smoc2 showed temporally distinct expression patterns (Figure 2). In particular, our results indicated that the expression of Smoc2 was very weak in the early embryonic stage in wild-type and Runx2 KO mice (Figure 2e and f). In contrast, Smoc2 was highly expressed in the skulls of wild-type mice, but it was not highly expressed in the skulls of Runx2 KO mice. Furthermore, Smoc1 was regionally restricted to the dorsal and ventral surface regions that lacked expression of Bmp4 and Bmp7. We are unsure which BMP ligands specifically regulate Smoc1 and Smoc2 expressions during development; it remains challenging to elucidate the relationships of specific BMP ligands with Smoc1 and Smoc2 expressions. We have mentioned these points in the Discussion while avoiding overinterpretation of our findings.

**Reply to Reviewer 1's point**
*The reviewer's comments has been sufficiently addressed. They now show the data requested for all of the genotypes and at the developmental stage requested. The data is well presented in Fig.S11. However, the new data needs a little more explanation and detail. For example, I think the new Smoc1(-/-)Smoc2(+/-) craniofacial phenotype is novel and requires some quantitation, similar to the micro-CT in supplementary figure 11. This is important as the Smoc1 null mice apparently do not have this phenotype and it suggests that there is compensation between the*

*Smocs, but this is insufficient for normal craniofacial development in a Smoc1-/-,Smoc2+/- genotype. They should also include a table indicating the penetrance of the phenotypes for each genotype, and at least show how many animals were observed with the phenotypes described. I could only find this for the calvaria phenotype but not the craniofacial defect.*

Reply: Because the bone calcification of several mutant mice was impaired at P0, the micro CT photographs of the mice were not particularly clear; we included them in this letter to allow assessment by the reviewer. We quantified both nasal bone to eye distance and length of mandibular bone in the mice as shown below; however, the differences were not statistically significant. If the reviewer feels that it is appropriate to incorporate these data in the manuscript, we are willing to include them as Supplementary Figures. In accordance with the reviewer's suggestion, we modified Supplementary Table S3 to include penetrance data regarding the calvaria morphogenesis and craniofacial anomaly phenotypes.

**Micro CT photographs of mice (all genotypes)**

*Smoc1*$^{+/-}$;*Smoc2*$^{+/-}$ mice were mated; the resulting mice were subjected to micro CT analysis at P0.

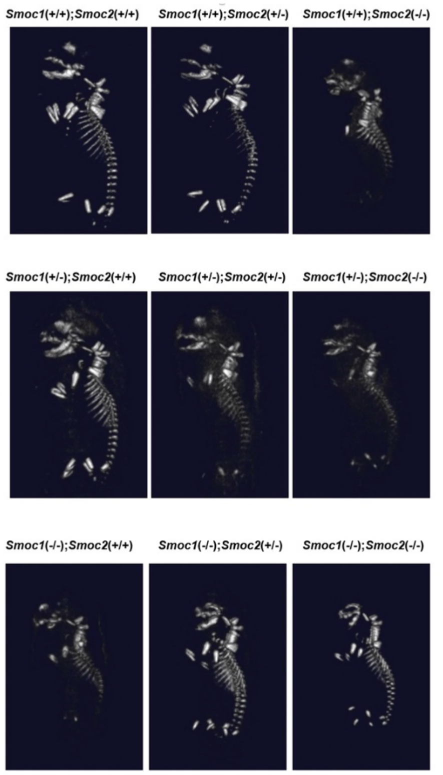


**Nasal bone to eye distance and length of mandibular bone**

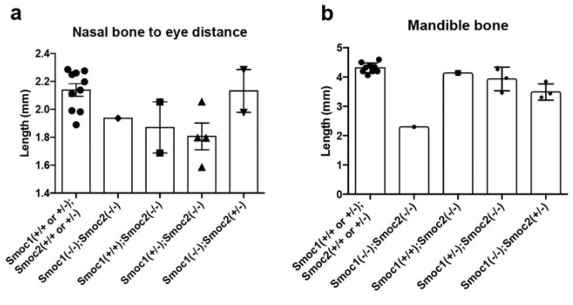


*Smoc1*$^{+/-}$;*Smoc2*$^{+/-}$ mice were mated; (a) nasal bone to eye distance and (b) length of mandibular bone were measured at P0. (n=1–10)

REVIEWERS' COMMENTS:

Reviewer #3 (Remarks to the Author):

I thank the authors for addressing my outstanding concerns.
